# Inverting family GH156 sialidases define an unusual catalytic motif for glycosidase action

**DOI:** 10.1038/s41467-019-12684-7

**Published:** 2019-10-23

**Authors:** Pedro Bule, Léa Chuzel, Elena Blagova, Liang Wu, Melissa A. Gray, Bernard Henrissat, Erdmann Rapp, Carolyn R. Bertozzi, Christopher H. Taron, Gideon J. Davies

**Affiliations:** 1 Department of Chemistry, University of York, York, YO10 5DD UK; 2 New England Biolabs, 240 County Road, Ipswich, MA 01938 USA; 3 Department of Chemistry, Stanford University, Stanford, CA 94305-4404 USA; 4 Architecture et Fonction des Macromolécules Biologiques (AFMB), Centre National de la Recherche Scientifique (CNRS, UMR7257), Institut National Agronomique (INRA, USC 1408) and Aix-Marseille Université (AMU), 13288 Marseille cedex 9, Marseille, France; 5 Max Planck Institute for Dynamics of Complex Technical Systems, Sandtorstrasse 1, 39106 Magdeburg, Germany; 6 glyXera GmbH, Leipziger Strasse 44–ZENIT, Magdeburg, Germany; 7 Howard Hughes Medical Institute, Stanford University, Stanford, CA 94305-4404 USA

**Keywords:** X-ray crystallography, Chemical biology, Carbohydrates, Enzyme mechanisms, Glycobiology

## Abstract

Sialic acids are a family of related sugars that play essential roles in many biological events intimately linked to cellular recognition in both health and disease. Sialidases are therefore orchestrators of cellular biology and important therapeutic targets for viral infection. Here, we sought to define if uncharacterized sialidases would provide distinct paradigms in sialic acid biochemistry. We show that a recently discovered sialidase family, whose first member EnvSia156 was isolated from hot spring metagenomes, defines an unusual structural fold and active centre constellation, not previously described in sialidases. Consistent with an inverting mechanism, EnvSia156 reveals a His/Asp active center in which the His acts as a Brønsted acid and Asp as a Brønsted base in a single-displacement mechanism. A predominantly hydrophobic aglycone site facilitates accommodation of a variety of 2-linked sialosides; a versatility that offers the potential for glycan hydrolysis across a range of biological and technological platforms.

## Abstract (web summary)

Sialidases are glycoside hydrolases that cleave sialosides. Here the authors define the 3-D structure, alone and in complex with products and inhibitors, of the CAZy family GH156 sialidase, EnvSia156, showing it displays a catalytical (β/α) 8-barrel domain distinct from other sialidases and allowing description of its inverting catalytic mechanism.

## Introduction

Sialic acids are a family of unusual keto-sugars (2-keto-3-deoxy-nononic acids) that have myriad roles in biology. The C5-amino version is the most well-known neuraminic acid, which is typically acetylated (Neu5Ac) or glycolated (Neu5Gc). They are most typically found capping glycan-chains on both glycoproteins and lipids and by virtue of their terminal positions they are keenly positioned to play major roles in protein–carbohydrate interactions and cellular recognition. Additional modification of the sialic acid core expands the sialic acid family, leading to sialic acids that can modulate or evade biological recognition^1,2^.

In mammalian cells, sialosides (typically α2-3, -6 or -8 linked) are perhaps most notable as a glyco-signature of cell type and health; interacting with sialic-acid-binding lectins termed Siglecs in diverse immune responses^3–6^. Sialic acids are also known as the recognition point that facilitates host cell invasion by numerous pathogenic viruses, such as influenza, certain parasites like *Trypanosoma cruzi*, and harmful bacteria, such as *Hemophilus influenzae, Streptococcus pneumoniae*, and *Pseudomonas aeruginosa*^7–15^. There is, therefore, considerable interest in the proteins that recognize sialic acids (both in the host and pathogens), and the enzymes that cleave and release sialic acids (sialidases and neuraminidases).

Sialidases have recently become of additional interest because of their application in precision glycocalyx editing, with applications in cancer immunotherapy^16^. Cell surface sialosides are harnessed by tumor cells to evade destruction by the immune system. Sialidases, chemically linked to anti-cancer antibodies, such as Trastuzumab, shave sialosides from the cancer cell surface preventing binding by immune-suppressing Siglecs. Targeting immune-modulating sialic acid enzymatically has thus gained new importance and has spurred efforts to discover new sialidases.

Sialidases, neuraminidases (EC 3.2.1.18) are glycoside hydrolases that cleave sialosides, typically acting in an “*exo*”-fashion at the sialic acid cap of glycan chains. At the sequence level, these enzymes have been classified into five of the CAZy sequence-based families^17^; 33, 34, 58, 83, and the recent GH156 family, described here. At the structural/mechanistic level, CAZy GH33, 34, and 83 are *exo*-sialidases, all displaying a similar six-fold β-propeller motif and all performing catalysis with net retention of anomeric configuration through the formation, and subsequent breakdown, of a covalent intermediate to a conserved active center tyrosine; elegantly revealed by the work of Newstead and colleagues^18^. These enzyme families are perhaps the best known and characterized, not least because they contain the influenza virus sialidases (CAZy GH34), which are the target for the anti-influenza drugs oseltamivir and zanamivir (Tamiflu and Relenza). CAZy family GH58 is an unusual *endo*-polysialidase family (EC 3.2.1.129), also displaying a six-fold β-propeller motif but which, in contrast to families 33, 34, and 83, acts with inversion of anomeric configuration. It cleaves within poly (α-2,8) sialic acid (PSA) polysaccharides mainly found in the capsule of some bacterial pathogens, like *Pasteurella haemolytica*, *Neisseria meningitidis*, and *Escherichia coli*, conferring them the ability to evade the host’s immune system^19–21^, and in the mammalian brain, where they are involved in several neurological processes including neural plasticity, neural–cell interaction, and growth^22–24^. Recently, the new CAZy family GH143 was created to classify a *B. thetaiotaomicron* enzyme capable of cleaving 2-keto-3-deoxy-d-lyxo-heptulosaric acid (Dha), a seven-carbon sialic acid analog, from rhamnogalactorunan-II (RGII)^25^. It is a bi-modular enzyme and it actually possesses two active centers with distinct activities: one β-l-arabinofuranosidase (EC 3.2.1.185) site that releases l-arabinofuranose from RGII chain D and a Dha-hydrolase site that subsequently cleaves chain D Dha from the main galacturonic acid chain. The Dhase site presents a slightly different fold from what is observed in other sialidases, with a five-fold β-propeller, but it retains the typical tyrosine–glutamate nucleophilic pair, implying it functions with a similar retaining catalytic mechanism.

Recently, as part of an effort to discover novel sialidases with potential biotechnological application, a family of *exo*-sialidases has been defined through isolation of sialidase activities from a freshwater hot spring environmental niche^26^. This family, termed CAZy family GH156, is unusual in that its members act with inversion of the anomeric configuration of the released sialic acid suggesting they have a distinct active-center geometry (implying a different 3-D topology for sialidase action). This enzyme was shown to act on a variety of (typically α2-3, α2-6 linked) sialic acid glycosides including complex N-glycans and O-glycan-linked sialic acids, in solution^26^. Here we describe the 3-D structure and catalytic center of the defining member, EnvSia156, of this sialidase family. We show, through ligand complexes with products and sialidase inhibitors, that the 3-D structure and active center are indeed an unusual arrangement, with family-conserved histidine and aspartate residues defining the catalytic mechanism that acts with inversion of anomeric configuration; facets not previously observed in the canon of literature for sialidases.

## Results and discussion

### Expression, activity, and oligomeric status of EnvSia156

**Fig. 1:**
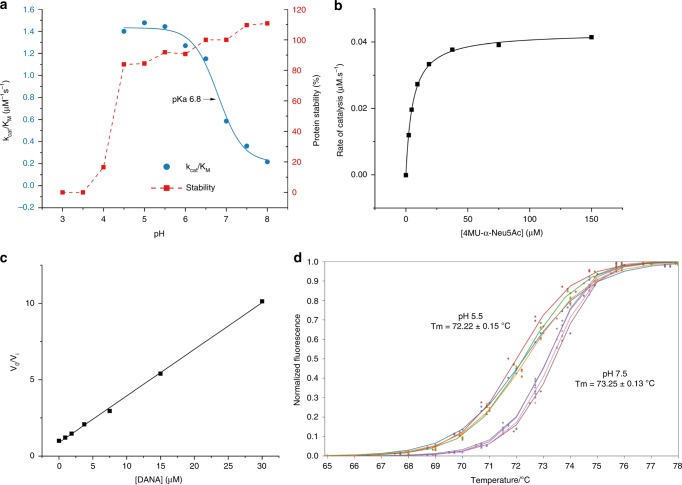
EnvSia156 activity and stability. **a** pH dependence of EnvSia156 using 4MU-α-Neu5Ac as substrate to monitor hydrolysis. Red squares show relative enzyme activity after incubation at different pH. Enzyme activity was assayed at pH 5.5 after preincubating EnvSia156 for 30 min at the several pH values. The rate obtained is shown as a percentage of the rate obtained at pH 7. EnvSia156 is unstable below pH 4 over the time period tested. Blue circles show the *k*_cat_/*K*_M_ measured at each pH by the substrate depletion method. The profile shows an optimal pH for hydrolysis of 5 with a p*K*_a_ of 6.8. Experiments were performed in triplicate. **b** Michaelis–Menten plot of 4MU-α-Neu5Ac hydrolysis by EnvSia156. Rate of catalysis was determined in triplicate for each 4MU-α-Neu5Ac concentration. **c** Plot of EnvSia156 *V*_0_/*V*_i_ against inhibitor concentration [DANA]. Reaction rate was determined by measuring the release of 4-methylumbelliferone (4MU) in the absence (*V*_0_) and presence (*V*_i_) of inhibitor. The 1/*K*_i_ value was derived from the slope of the best fit line. Experiments were performed in triplicate. **d** DSF curve of EnvSia156 (12.5, 25, 50, and 100 nM) at pH 5.5 and 7.5. Fluorescence measurements were performed in triplicate. Source data are provided as a Source Data file

Gene cloning, expression, and purification of recombinant EnvSia156 was performed as previously described by Chuzel et al. ^26^. The kinetics of the purified recombinant protein used for crystallization were determined, using the substrate depletion method^27^, with 2′-(4-methylumbelliferyl)-α-d-*N*-acetylneuraminic (4MU-α-Neu5Ac) as a substrate, at pHs between 3 and 8 (Fig. 1a). The enzyme shows instability below pH 4.5, but the alkaline limb of the pH profile may be fitted with a single ionization to give an approximate optimal pH of 5 with a p*K*_a_ of 6.8 for the descending curve. At pH 5, the enzyme yielded a *k*_cat_ of 4.28 ± 0.06 s^−1^ and *K*_M_ = 5.58 ± 0.05 µM (Fig. 1b) putting EnvSia156 amongst sialidases with the highest catalytic efficiency reported to date^28,29^. An initial screen of potential inhibitors suggested that EnvSia156 was inhibited by *N*-acetyl-2, 3-dehydro-2-deoxyneuraminic acid (DANA) (Fig. 1c), but not by Siastatin B. An apparent inhibitor constant (*K*_i_) of 3.17 ± 0.41 µM was determined for DANA at low substrate concentration ([*S*] ≪ *K*_M_) using 4MU-α-Neu5Ac.

To ascertain the oligomeric status of EnvSia156, SEC-MALLS experiments were performed, indicating that EnvSia156 was a dimer in solution with an estimated absolute molecular mass of 119.2 kDa. This closely approximated the predicted molecular mass of an EnvSia156 dimer (117.4 kDa) calculated using the ProtPram tool^30^ (Supplementary Fig. 1). The thermal stability was assessed by differential scanning fluorimetry (DSF) at pH 5.5 and pH 7.5, and the obtained *T*_m_ values were 72 and 73 °C, respectively (Fig. 1d). This is compatible with the average reported temperature of 60 °C in the ecological niche from which the enzyme originates. The slight increase in thermal stability at pH 7.5 is in agreement with the pH stability curve.

### The structure of EnvSia156 defines an unusual sialidase fold

To explore the 3-D structure and molecular determinants supporting the unique catalytic mechanism described by Chuzel et al. ^26^, the structure of EnvSia156 was determined on its own and in complex with *N*-acetylneuraminic acid (Neu5Ac), *N*-glycolylneuraminic acid (Neu5Gc), 2-keto-3-deoxy-d-glycero-d-galacto-nononic acid (KDN), and the inhibitor DANA. The absence of related 3-D structures dictated the need for a selenomethionine approach to phasing, so a selenomethionine derivative was crystallized and data collected at three different wavelengths (selenium peak, inflection, and high-energy remote). The resulting refined SeMet structure was subsequently used as the starting model for refinement of unliganded EnvSia156 and complexes with 20 mM Neu5Ac, Neu5Gc, DANA, KDN. Although all collected datasets were highly anisotropic, which ultimately had a negative impact on refinement statistics, they resulted in good quality 2*F*_o_−*F*_c_ maps, with clearly defined amino acid sidechains and ligands easily identifiable in the difference maps. The best model was obtained from a crystal of EnvSia156 with bound Neu5Ac (EnvSia156Neu5Ac), at a resolution of 2.0 Å. Data collection and refinement statistics for all structures are summarized in Supplementary Table 1.

**Fig. 2:**
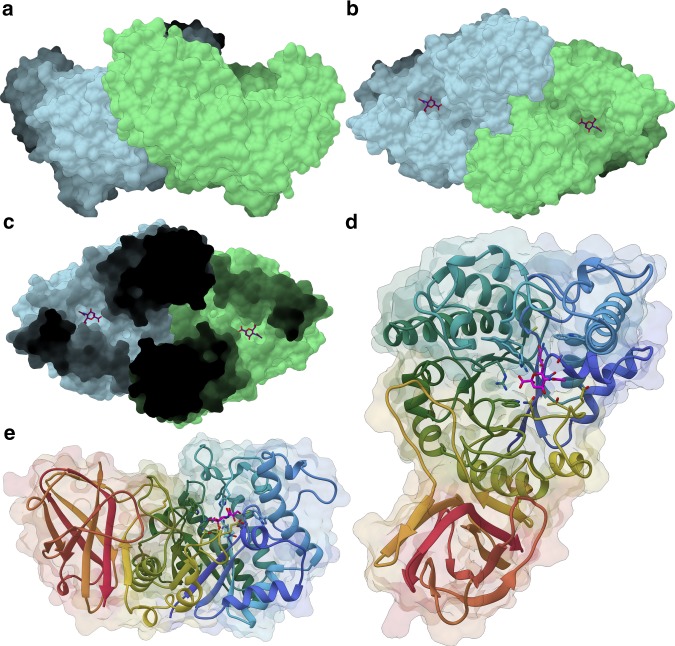
The 3-D structure of EnvSia156. **a**, **b** Van der Waals’ surface of a EnvSia156 homodimer with one unit colored in light blue and the other in light green. **c** “Top-down” view of the dimer with depth shading highlighting the continuous groove connecting the two binding sites. **d**, **e** Ribbon representation of the structure of EnvSia156 with rainbow coloring (N-terminus in blue, C-terminus in red) showing the (β/α)_8_-barrel fold of the catalytic module and the eight stranded β-sandwich C-terminal module. A purple colored Neu5Ac molecule in stick representation can be seen bound in the active center of each unit present in panels **b**–**e**

Two copies of the protein were present in the asymmetric unit, each composed of two distinct domains (Fig. 2a, b). The two copies appear to be related by non-crystallographic symmetry resulting in a dimer, discussed below in light of the interface analysis and SEC-MALLS results. The catalytic domain (residues 6–376) comprises a complete (β/α)_8_-barrel fold while the C-terminal domain (residues 377–502) consists of an eight-stranded β-sandwich (Fig. 2d, e). The functional side of a barrel enzyme is normally present on the “N-terminal” face of the barrel, defined by the βα-loops (loops with a β-strand on the amino end and an α-helix on the carboxy-terminus end), which are therefore longer than the purely structural αβ-loops^31^. That considered, EnvSia156 βα-loops are still remarkably elongated. A particularly extensive loop is formed by residues 50–85, connecting β-strand 2 to α-helix 2, which is itself an unusually large helix (residues 86–119) and one of the most distinctive features of EnvSia156 structure. The C-terminal β-sandwich domain is formed by eight antiparallel β-strands organized into two β-sheets, with one sheet formed by strands 1, 3, 6, and 8 and the other by strands 2, 4, and 7, while strand 5 is shared between the two faces. The larger face of the β-sandwich folds on itself as the loop connecting β-strands 5 and 6 bends towards the catalytic domain and interacts with the long 7th βα-loop of the barrel, contributing for structural stabilization. The first β-strand of the C-terminal domain is interrupted by a long loop (residues 382–399) that likely plays a role both in overall structural stabilization and dimer assembly. With *B*-factors values ranging from 16 to 20 it is one of the most rigid features in the structure, which is unusual for a large loop. This loop makes several important hydrophobic interactions with both its dimeric counterpart and the catalytic domain, contributing to the formation of a continuous cleft connecting the active sites of the two units in the dimer (Fig. 2c). According to PDBsum^32^, 20% of the residues establishing non-bonded contacts between the two units are located in this loop, highlighting its role in the dimerization process. An assembly analysis with PISA calculated an interaction surface area between the two monomers of 1725 Å^2^ with a predicted solvation energy gain (Δ*G*) of –13.2 kcal/mol, suggesting that the observed homodimer formed by the two copies present in the asymmetric unit is the natural biological assembly of EnvSia156.

The structure of EnvSia156 is clearly different from all the known families of both exo-sialidases and endo-sialidases which, despite some differences regarding accessory domains, substrate-binding clefts or catalytic residues; all possess a six-bladed β-propeller topography^33–37^. A structural similarity search on DALI server^38^ using the catalytic domain of EnvSia156 (residues 6–375) returns Cwp19 “functional” region as the closest structural homolog (PDB code 5OQ2, *z* score of 27.2 and rmsd of 3.4 Å over 299 aligned residues^39^) with 14% identity between aligned regions. Cwp19, produced by *Clostridium difficile* during stationary phase to induce autolysis, has been recently described as a lytic transglycosylase with activity on peptidoglycan^40^, but beyond the superficial fold resemblance there is no conservation of active center residues and indeed the ligand complexes of GH156 (vide infra) clash with Cwp19 main chain following overlap.

The C-terminal β-sandwich domain (residues 376–502) shows distant homology to a domain from a *Bacteroides uniformis* uncharacterized family 86 glycoside hydrolase, possessing agarase activity (PDB code 5TA5, *z* score of 11.6 and rmsd of 2.1 over 101 aligned residues). It is also distantly related to a CBM4 domain appended to a porphyranase produced by *Bacteroides plebeius*, similarly classified as a GH86 (PDB code 4AW7, *z* score of 9.7 and rmsd of 2.0 Å over 102 aligned residues). Both are bacteria found in the human gut microbiome^41,42^ thought to have acquired the GH86 genes by horizontal gene transfer with sea dwelling organisms. Spatially equivalent surface aromatics (Supplementary Fig. 2) may imply a CBM-like role for the β-sandwich domain of EnvSia156, but that remains to be established.

### Complexes and enzyme variants define the active-center and mechanism

Having ascertained that EnvSia156 indeed displays an unusual sialidase fold, we next sought to define the active center of this family. A sequence comparison with the closest primary structure homologs found in GenBank using the BLASTP tool (Supplementary Fig. 3), shows that areas with the highest degree of conservation are mostly found within the β-strands of the (β/α)_8_-barrel or just after it, on the “N-terminal” face of the (β/α)_8_-barrel.

**Fig. 3:**
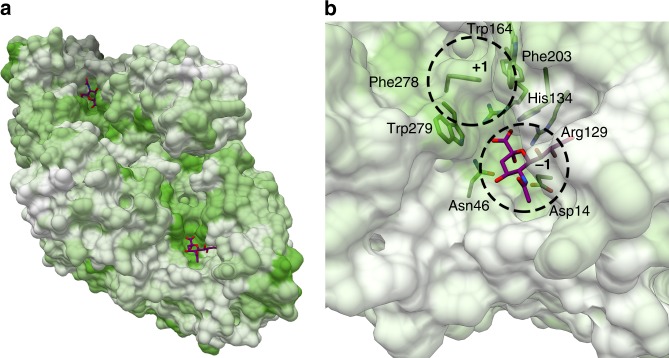
EnvSia156 homology and binding subsites. **a** Birdseye view of an EnvSia156 dimer with the van der Waals’ surface colored according to sequence conservation, calculated by aligning sequences (ClustalOmega) with the closest primary structure homologs found in GenBank using the BLASTP tool. The color pattern matches the alignment used to render the structure (Supplementary Fig. 3), with darker shades of green corresponding to full conservation and white to no conservation. **b** Detailed view of the catalytic site of EnvSia156 with putative substrate coordinating residues in a stick representation, colored according to sequence conservation. The −1 and putative +1 binding sub-sites are highlighted with dashed circles

**Fig. 4:**
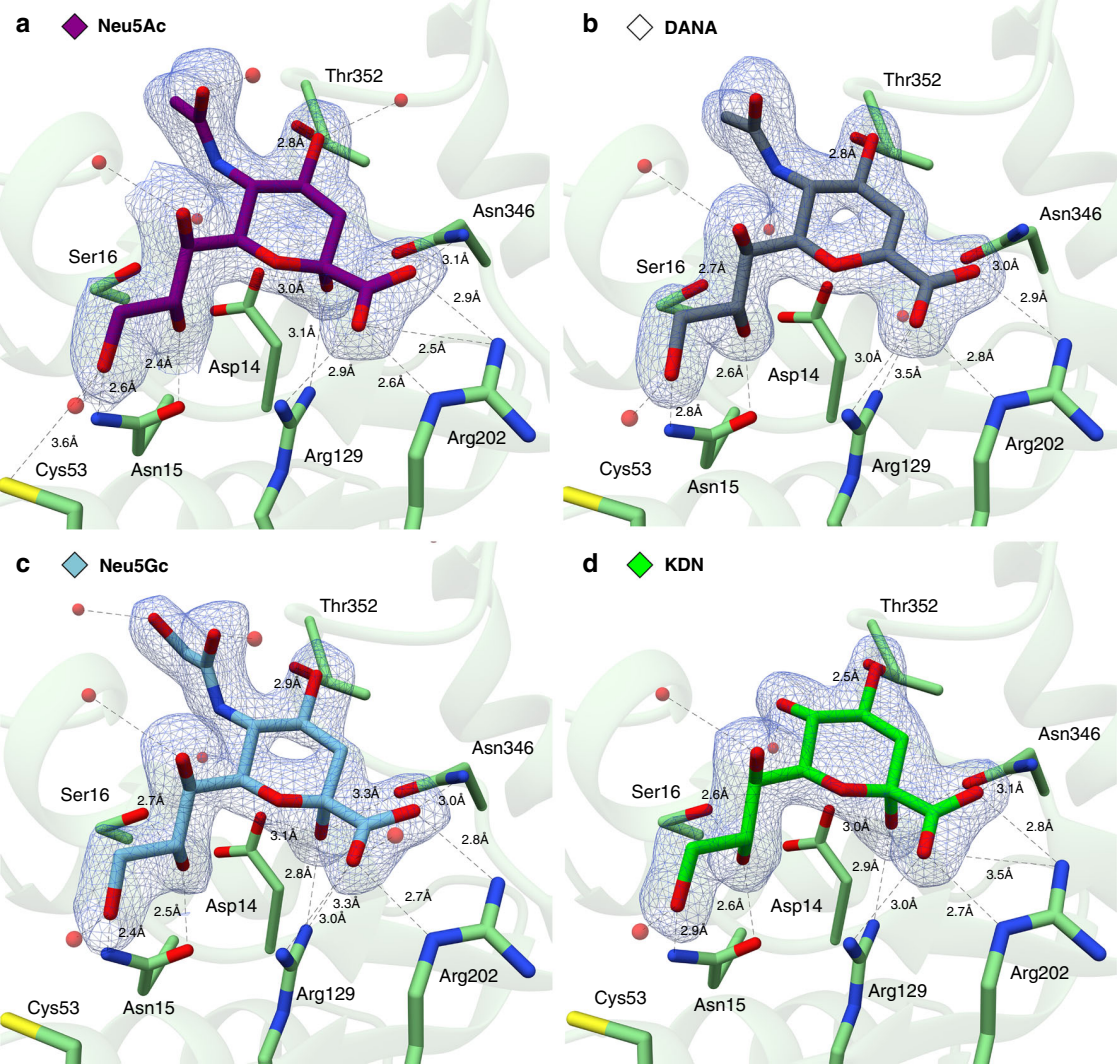
Crystal structures of EnvSia156–ligand complexes. The four panels show a detailed view of the crystallized EnvSia156–ligand complexes within the active center. The enzyme residues making hydrogen-bond contacts (black dashed lines) with the ligands are displayed in stick representation. The ligands are surrounded by a mesh representation of the Refmac5 maximum-likelihood σ_A_–weighted 2*F*_o_−*F*_c_ electron density map contoured at 1*σ* (0.46 electrons/Å^3^), and are colored purple (**a** Neu5Ac), gray (**b** DANA), light blue (**c** Neu5Gc), and green (**d** KDN). Water molecules are colored red

By coloring the structure according to homology, using the same color scheme as in the alignment, it is possible to identify a highly conserved pocket on the “N-terminal” face of the (β/α)_8_-barrel that sits at both ends of the continuous groove formed by the dimer (Fig. 3a, b). Considering its position and conservation, this site is a likely candidate to represent the EnvSia156 catalytic center. Classic retaining *exo*-sialidase enzymes have an active center characterized by a triad of arginines that stabilize the carboxylate of sialic acid, three catalytic residues—a glutamic acid, a tyrosine, and an aspartic acid—and an hydrophobic pocket that accommodates the C5 moiety^43^. The identified conserved pocket indeed shows the presence of three 100% conserved arginines (Arg129, Arg 202, and Arg246), hinting at a sialoside-binding region, although one of them, Arg246, seems to be too buried to be able to interact with the carboxylate moiety of sialic acid. EnvSia156 also displays several highly conserved hydrophobic residues forming a pocket that could potentially accommodate the aglycone moiety of the sialoside substrates. To better define, the active center residues, 3-D structures were determined with the product Neu5Ac and with the inhibitor DANA (*K*_i_ 3 μM). Electron density was clear for all ligands permitting definition of the key elements of sialic acid recognition for this family (Fig. 4).

The structures with Neu5Ac show the β-anomer (equating to a product complex following catalytic attack on an α-sialoside with inversion) of the ligand interacting with EnvSia156 at the predicted catalytic center based on sequence homology, through 13 hydrogen bonds (with 8 more mediated by water molecules) and several hydrophobic interactions (Fig. 4a). The –1 subsite, which accommodates the sialic acid monosaccharide, shows several differences from what is commonly observed in other sialidases. It is possible to identify the characteristic triad of residues coordinating the carboxylate group, although it consists of two arginine side-chains (Arg129 and Arg202) and one asparagine (Asn346), instead of the three arginines displayed in conventional sialidases. Invariant Asp14 lies adjacent to the (anomeric) C2 carbon and makes a hydrogen bond with the hydroxyl group. Thus, Asp14 is the most likely candidate to act as a general catalytic base residue (discussed further below).

**Fig. 5:**
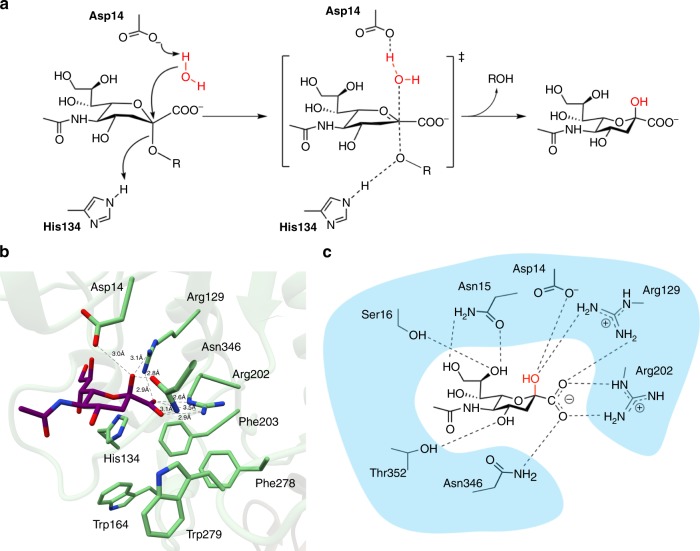
EnvSia156 proposed catalytic mechanism. **a** Proposed inverting mechanism for EnvSia156 with Asp14 acting as the general catalytic base and His134 as the acid catalyst. **b** Structure of the EnvSia156–Neu5Ac complex highlighting the two putative catalytic residues (Asp14 and His134), the carboxylate-coordinating triad (Arg129, Arg202, Asn346) and the hydrophobic pocket that possibly constitutes the +1 binding site (Trp164, Phe203, Trp279, Phe278). **c** Schematic representation of the Neu5Ac bound to EnvSia156 −1 binding sub-site, showing all the hydrogen bond interactions between both molecules

Another major difference between EnvSia156 and other sialidases is the absence of a hydrophobic pocket that accommodates the C5 moiety of sialic acids. Rather, the more open structure of the −1 sub-site orientates the sialic acid in such way that the functional group at the C5 position is pointing away from the enzyme. The structure of EnvSia156 with Neu5Ac shows a single discrete non-bonded contact between Gln351 and the acetamide methyl group and one water-mediated hydrogen bond between N5 and Asp14/Ser16. This suggests that EnvSia156 can tolerate different C5 moieties and explains why, while showing some preference for Neu5Ac, it can also hydrolyze terminal Neu5Gc^26^. To test this supposition, the structure of EnvSia156 in complex with KDN was obtained, showing that this sialic acid with a simple hydroxyl group at the C5 position can also be accommodated (Fig. 4d). All complex structures show the protein forming hydrogen bonds with all three hydroxyl groups in the glycerol chain, although the residues involved are not as conserved as those mentioned above. Tyr20 and Tyr135 bind with O7 via a water molecule, Ser16 and Asn15 coordinate O8, and O9 contacts Asp132 and His134 via a water molecule, while binding directly to Asn15 and Cys53. This is not a common feature in sialidases and suggests that the glycerol group is a key feature for substrate recognition. This could also explain why the activity of EnvSia156 is not inhibited by Siastatin B. Curiously, both the open structure around the C5 moiety and tight glycerol chain coordination have been previously reported on a viral hemagglutinin-neuraminidase (HN)^33^. In classic sialidases, it is believed that the hydrophobic pocket accommodating the C5 functional group fixates one end of the sialic acid and allows the ring distortion that exposes the anomeric carbon. It could be that, in the case of EnvSia156 and the paramyxovirus HN, this hinge-like mechanism relies on the immobilization of the glycerol chain rather than the C5 moiety. The C4 hydroxyl group being coordinated by Thr352 and Trp279 (via a water-mediated bond). EnvSia156 acts, in solution at least (see below) on a variety of α2-3, and α2-6 linked sialic acid glycosides including complex N-glycans and O-glycan-linked sialic acids. The diversity of structures accepted as leaving groups suggests a more open and less hydrogen-bonded environment and indeed we observe a hydrophobic platform, adjacent to the −1 sub-site. Four highly conserved aromatic residues, namely Trp164, Phe203, Phe278, and Trp279, forming a hydrophobic pocket that could possibly accommodate the aglycone moiety in the +1 binding site (Figs. 3b and  5).

In the classic retaining mechanism of exosialidases, the carboxylate group of a glutamate forms a hydrogen bond with the hydroxyl group of the catalytic tyrosine, increasing its nucleophilic character, which in turn attacks the anomeric carbon to form a covalent glycosyl-enzyme intermediate. An aspartate functions as the acid/base residue donating the proton on this initial step and then facilitating the nucleophilic attack of the glycosyl-enzyme intermediate^44^. Contrastingly, but as expected for an inverting enzyme, none of the signature retaining sialidase residues are identifiable in the EnvSia156 active site.

EnvSia156 is an inverting enzyme^26^ which demands two catalytic residues: a Brønsted acid to protonate the leaving-group and a Brønsted general base to activate the nucleophilic water molecule for nucleophilic attack^45–48^. Asp14 is ideally placed to assume the role of general base, through its position on the “beta” face and its direct interaction with the product OH in the Neu5Ac complex. Furthermore, in the DANA complex, Asp14 coordinates a single water molecule below the anomeric C2 carbon, a position that mimics what would be seen in a substrate complex. On the “alpha” face side of the sugar, His134 could potentially act as the Brønsted acid catalyst that donates the hydrogen to the leaving group (Fig. 5).

To test the hypothesis that Asp14 and His134 are key catalytic residues, Asp14 and H134 variants were created. Using the activated substrate analog 4MU-α-Neu5Ac, all Asp14 mutants were inactive, consistent with the role of Asp14 as general base. In contrast, His134 variants suffered a significant drop (~30%) in activity (Supplementary Fig. 4a), consistent with the reduced need for protonic assistance to liberate the good leaving group present on the activated sugar analog substrate. Kinetics using the H134A variant at pH 5, yielded a *k*_cat_ of 24.6 ± 0.02 s^−1^ and *K*_M_ = 305.52 ± 37.8 µM. If H134 functioned as a general acid, we would predict that it would be less active on less-activated substrates having higher p*K*_a_ leaving groups. This was confirmed by incubating a procainamide-labeled α-2,3-sialyl-lactose substrate at pH 5.5, for 1 h at 37 °C and by detecting both product and substrate by UPLC. Using these reaction conditions, an equivalent amount of WT enzyme was able to hydrolyze 77.5 ± 1.8% of the substrate, whereas no activity was detected for the H134A-mutant enzyme (Supplementary Fig. 4b). This observation strongly supports the notion that H134 acts as a general acid during catalysis of natural substrates. An analogous Asp-His catalytic dyad has been similarly proposed for members of the GH117 family^49,50^. They consist of agarolytic enzymes that act through a single-displacement inverting mechanism, similar to the one here proposed for EnvSia156, although in this case the enzymes display a different beta-propeller fold^49,50^.

Despite substantial efforts, we were unable to obtain a Michaelis complex of EnvSia156 using the His134, Asp14 (or double) variants. So, in the absence of a true Michaelis complex for EnvSia156, we overlaid the EnvSia156Neu5Ac structure with the Michaelis complex of the *T. cruzi* *trans*-sialidase with α-2,3-sialyl-lactose (PDB 1S0I). The Michaelis complex overlay shows how the carboxylate coordinating triad and a binding pocket that accommodates the aglycon moiety may contribute to distort the sialic acid ring exposing the anomeric carbon to nucleophilic attack^35^. When overlaying this structure with that of EnvSia156Neu5Ac it is possible to speculate that a similar process occurs, with the carboxylate held in a pseudo-equatorial orientation by Arg129, Arg202, and Asn346, and the aglycone fitting into the pocket formed by Trp164, Phe203, Phe278, and Trp279, forcing the glycosidic bond into a pseudo-axial orientation. This would likely allow a water molecule to position itself behind the glycosidic bond. In fact, the structure of EnvSia156 in complex with the inhibitor DANA (EnvSia156DANA), which simulates the planar transition state due to its *sp*^2^ hybridized C2, shows a water molecule occupying the same relative position to the anomeric carbon as the hydroxyl nucleophile of the catalytic tyrosine in the *T. cruzi* *trans*-sialidase, while Asp14 replaces its glutamate-activating partner (Supplementary Fig. 5). A prior study by Newstead et al. showed that a retaining sialidase could be converted into an inverting sialidase by mutation of the catalytic tyrosine and having a water molecule act as the nucleophile^51^. Thus, we speculate that the inverting catalytic mechanism of EnvSia156 likely involves Asp14-activation of an analogous nucleophilic water molecule that attacks the anomeric carbon, with His134 donating a proton to the leaving group (Fig. 5).

### Can GH156 be used for glycocalyx engineering?

Considering that GH156 defines a sialidase different to all known neuraminidases and was active in solution on diverse sialic acids, we were keen to see if it could be used for engineering of cell-surface glycosides. When activated, certain Siglec receptors present on immune cells lead to the suppression of cell-mediated immunity. Sialic acid glycans present on the glycocalyx of mammalian cells can activate these receptors and help the immune system to identify them as self. Certain cancer types have evolved towards exploiting this system by coating themselves with a large number of sialylated glycans, which allows them to evade the host immune system, both cellular and antibody dependent. Indeed, hypersialylation in cancer is associated with poor prognosis and decreased immunogenicity^52–54^. Previous studies have shown that by conjugating sialidases with antibodies it is possible to direct their activity towards the specific desialylation of tumor cells, exposing them to numerous immune pathways^16^. At first sight, EnvSia156 seems to be particularly suited for this purpose as it is capable of binding different sialic acids and has shown to be active in a variety of sialosides^26^. Given the potential biomedical applications of sialidases, and high specific activity and the diversity of ligands (α2-3, and 2-6-linked sialic acid glycosides including complex N-glycans and O-glycan-linked sialic acids^26^) in solution, we wanted to test the ability of EnvSia156 sialidase to remove sialic acids from living cells. The K562 cell line derived from chronic myelogenous leukemia was used as a model cell line and treated with EnvSia156 (4.25 μM) to detect sialic acid loss compared to PBS-treated cells. As a positive control for desialylation, we also treated cells with 0.2 μM of *Vibrio cholerae* sialidase, which has been previously shown to cleave sialic acids from cell surfaces. Cell-surface sialic acids were analyzed by flow cytometry detecting lectin binding with *Sambucus nigra* lectin (SNA, which binds primarily to α-2,6 sialic acids) and *Maackia amurensis* lectin II (MAL II, which binds preferentially to certain α-2,3-linked sialic acids). Interestingly, at these concentrations EnvSia156 does not appear to remove MAL II ligands, and only slightly diminishes SNA binding to the cell surface (Supplementary Figs. 6 and 7).

Given the catalytic prowess of EnvSia156 towards aryl sialosides, we were surprised not to see better action on a cellular model. We have also tested the binding of an inactive (catalytic base D14A) mutant variant of EnvSia156 using a printed glycan array consisting of 585 glycans (Supplementary Table 2). Binding to all glycans was very low with both 5 and 50 μg/mL of protein. By increasing the concentration to 200 μg/mL, significant binding, albeit still relatively weak, was detected for the sialoside Neu5Aca-Sp11 (Supplementary Fig. 8). The signal detected for the remaining glycans are likely the result of unspecific interactions, as Neu5Aca-Sp11 is the only consistent binder across all protein concentrations. Considering the catalytic activity reported here and by Chuzel et al.^26^ on labeled sialyl-lactose and sialyl-lactosamine, we were surprised that no binding was detected for any of the terminal Neu5Ac-α-(2,6)Gal and Neu5Ac-α-(2,3)Gal glycans. The general weak binding in the glycan array appears to corroborate the weak performance on the K562 cell line, suggesting that some factor, beyond simple catalytic efficiency, is necessary for efficient glycocalyx engineering. Perhaps the presence of a true lectin/CBM domain is essential to target the enzyme to cell-surface glycans (and the immobilized glycans on the array slide), as it is in the case of the *Vibrio* sialidase, for example.

By virtue of its terminal position in protein-linked and lipid-linked glycans, sialic acid intimately participates in cellular recognition in both health and disease. Sialidases play key roles in health, but also in the environmental degradation of sialosides (of their diverse types). Considering the potential for medical, dietary, and biotechnological applications of sialidases, there is a continuing interest in defining new sialidase templates for application. As part of a screening effort to examine sialidases from extreme environmental niche, where the role of sialic acids is poorly understood, EnvSia156 (the defining member of the GH156) was isolated from a freshwater hot spring^26^. Here we show that GH156 sialidases define a distinct paradigm for sialidase action with His and Asp acting as Brønsted acid and base, respectively, in an inverting mechanism that is unique amongst all *exo*-sialidases described to date. We also show that this sialidase presents broad ligand specificity and high catalytic efficiency, meaning it can serve as a template for a variety of applications where high specific activity is required.

## Methods

### Gene expression and protein purification

The EnvSia156 gene was PCR-amplified from a fosmid clone with the addition of a C-terminal hexahistidine tag. The DNA fragment was inserted into the Ptac promoter expression vector pJS119K^55^. Primers were designed using the NEBuilder assembly tool (Supplementary Table 3). PCR products were assembled with the NEBuilder HiFi DNA Assembly Cloning Kit (New England Biolabs, Ipswich, MA) following the manufacturer instructions. The expression construct was verified by Sanger sequencing. EnvSia156 was expressed in *E. coli* NEB Express (New England Biolabs, Ipswich, MA). Expression was performed by addition of IPTG to 0.4 mM and incubation overnight at 18 °C. Cells from a 1 L culture were harvested by centrifugation at 15,000 × *g* for 10 min at 4 °C and resuspended in 30 mL of 20 mM sodium phosphate, pH 7.4, 500 mM NaCl, and 20 mM imidazole buffer prior to lysis with a TS Benchtop Series cell disruptor (Constant Systems Limited, Daventry, UK) at 32 kPsi. EnvSia156 was purified on a 5 mL His-Trap^TM^ FF column (GE Healthcare, Little Chalfont, UK). The bound EnvSia156 was eluted in a 20-column volume gradient of 20 mM sodium phosphate, pH 7.4 containing 500 mM NaCl and 0–500 mM imidazole. Fractions containing pure protein were pooled and dialyzed against 20 mM sodium phosphate, pH 7.4, containing 500 mM NaCl and 1 mM EDTA. Expression of seleno-methionine EnvSia156 derivative was performed in *E. coli* auxotrophic T7 Express Crystal strain background (New England Biolabs, Ipswich, MA) using the growth conditions described by Ramakrishnan et al.^56^ SeMet labeled cells were harvested by centrifugation at 4500 × *g* for 25 min at 4 °C. Cell paste from the 12 L culture was resuspended in 240 mL of 20 mM sodium phosphate, pH 7.4, 500 mM NaCl, 20 mM imidazole buffer and lysed using a TS Benchtop Series cell disruptor (Constant Systems Limited, Daventry, UK) at 32 kPsi. Purification of the SeMet-labeled sialidase-6His was performed in two steps. First, sialidase-6His was bound to a 5 mL His-Trap™ FF column (GE Healthcare, Little Chalfont, UK) and eluted with a 100 mL 0 to 500 mM imidazole gradient (in 20 mM sodium phosphate, pH 7.4, 500 mM NaCl). Fractions (3 mL) containing partially pure protein were pooled and the resulting solution (63 mL) was concentrated to 2 mL using Vivaspin 20, 30,000 molecular weight cut off tubes (Sartorius, Göttingen, Germany). Second, gel filtration chromatography was performed using a HiPrep 16/60 Sephacryl S-200 high-resolution column (GE Healthcare, Chicago, IL) and 50 mM Tris, pH 8.5, 200 mM NaCl buffer. The sample was prepared by diluting 1 mL of concentrated sialidase-6His with 1 mL of the column buffer and injecting it onto the column with a 2 mL sample loop. To help the identification of the catalytic residues D14A and H134A, mutants were generated both on the original gene (PCR-amplified from the fosmid clone) or by performing site-directed mutagenesis PCR on a pET29a vector containing the synthesized (Genscript) wild type EnvSia156 gene (Supplementary Table 4), using the primers shown in Supplementary Table 3. No differences exist between the amplified and synthesized clones. The synthesized gene and mutant derivatives were generated for ease of access purposes.

### Crystallization of EnvSia156

EnvSia156 at 14.6 mg/mL was tested against a range of commercial crystallization screens with the addition of 5 mM TCEP to every condition. The first crystals found consisted in stacks of plates and were used to create a seed stock. Optimization screens were set with and without seeds. Well diffracting single crystals were obtained by the sitting drop diffusion method at 293 K with 0.8 M sodium formate, 15% PEG 4000, 0.1 M sodium acetate, pH 6.0 and with 0.2 M MgCl_2_ 15% PEG 4000, 0.1 M sodium citrate, pH 5.6 using a protein per well ratio of 1:1. Seleno-methionine EnvSia156 at 21 mg/mL was crystallized using a similar protocol. The best diffracting crystals were obtained in 0.2 M lithium sulfate, 15% PEG 4000, 0.1 M sodium acetate, pH 6.0 using a protein per well solution ratio of 1:1. Crystals were cryoprotected in well solution with 25% glycerol and flash cooled in liquid N_2_ for data collection. Protein concentration was determined in an Epoch microplate spectrophotometer (Biotech), using a calculated extinction coefficient of 124,705 M^−1^ cm^−1^.

### Substrate and inhibitor complexes

EnvSia156 complexes were generated by co-crystallization with 20 mM Neu5Ac, Neu5Gc, DANA, or KDN. Initial screens and optimizations were performed using the same protocol described for the apo crystals. Ligands were introduced by addition of 0.2 µL of 100 mM stock solution in water to a 0.8 µL drop with a protein/well solution ratio of 1/1. Well-diffracting single crystals were obtained with 0.2 M potassium bromide, 15% PEG 4000, 0.1 M sodium acetate, pH 6.0 (DANA), 0.15 M potassium thiocyanite, 20% PEG 1500, 0.1 M sodium acetate, pH 6.0 (KDN), 0.8 M sodium formate; 12% PEG 4000; 0.1 M sodium acetate, pH 6.0 (Neu5Ac and Neu5Gc).

### 3D structure solution

The EnvSia156 SeMet derivative crystal structure was solved by multiple anomalous dispersion (MAD). Data were collected at beamline I04 of Diamond Light Source at the selenium peak, inflection, and high-energy remote wavelengths (0.97946, 0.97965, and 0.97873 Å), processed with DIALS^57^, reduced with Aimless^58^ and phased with the Crank2 pipeline^59^. The initial model was subsequently subjected to alternating rounds of manual building and refinement with Coot^60^ and REFMAC5^61^. The SeMet model was then used to solve the native apo and ligand crystal structures by molecular replacement. Data for the apo, NeuGc, and Neu5Ac complex crystals were collected at beamline I04 of the Diamond Light Source, while data for the remaining crystals were collected at beamline IO4–1 of the same synchrotron. All datasets were processed with DIALS, reduced with Aimless and phased with PhaserMR^62^. The obtain models were refined as described above. Ligand coordinates were built using JLigand^63^. wwPDB Validation Service^64^ was used to validate the structures before deposition in the PDB. 3D structure figures were generated using UCSF Chimera^65^.

### Enzyme activity and inhibition assays

Kinetic studies of EnvSia156 were performed at room temperature in flat-bottom 96-well opaque black plates (Thermo Scientific, Waltham, MA) by monitoring 4MU fluorescence at *λ*_ex_ = 365 nm and *λ*_em_ = 445 nm in a CLARIOstar microplate reader (BMG Biosciences). A standard curve was determined for each individual assay in order to convert measured RFU to 4MU concentration. All assays were performed in triplicate. The optimal pH for enzyme activity was determined by incubating 140 nM EnvSia156 with 2 mM of 4MU-α-Neu5Ac for 5 min at room temperature in a pH range of 3–8 (200 mM citrate/phosphate buffer, 0.1% BSA) and stopping the reaction by adding 1 M sodium carbonate buffer at pH 10.4.

Kinetic assays were performed in a total volume of 100 µL buffer (200 mM citrate/phosphate pH 5, 0.1% BSA) with 10 nM EnvSia156. The reaction was initiated by the addition of 50 µL of 4MU-α-Neu5Ac to the enzyme for a final substrate concentration range of 1.17–100 µM.

All data were processed using Origin (OriginLab). Rates were calculated by linear fit of 4MU concentrations measured between 0 and 48 s and kinetic parameters were determined using the Michaelis–Menten equation as follows:

1 $$v = \frac{{V_{{\mathrm{{max}}}}\left[ S \right]}}{{K_{\mathrm{{M}}} + \left[ S \right]}}$$

Inhibition assays were carried out in a total volume of 100 µL buffer (200 mM citrate/phosphate pH 5, 0.1% BSA) with 10 nM EnvSia156. The reaction was initiated by the addition of 50 µL of 4MU-α-Neu5Ac to 50 µL of a solution containing inhibitor and EnvSia156 for a final substrate concentration of 1 µM. Inhibitor concentrations used ranged from 0.94 to 30 µM. 4MU release was recorded continuously over a period of 300 s. Rates were determined in both the presence and absence of inhibitor. The *K*_i_ value was determined using the following equation:

2 $$\frac{{v_0}}{{v_{\mathrm{{i}}}}} = \frac{1}{{K_{\mathrm{{i}}}}}\left[ I \right] + 1$$

where *v*_0_ and *v*_i_ are the rates of catalysis in the absence and presence of inhibitor, respectively. Under conditions where [*S*] ≪ *K*_M_, the fractional decrease in rate thus yields the *K*_i_ for a competitive inhibitor. A plot of *v*_0_/*v*_i_ against inhibitor concentration yields a gradient of 1/*K*_i_, with an intercept of 1^66^.

Measurements of *K*_cat_/*K*_M_ were carried out by the substrate depletion method^27^ in a pH range of 3–8. Data were plotted as a function of pH and fitted with the following four-parameter logistic regression as follows:

3 $$\frac{{K_{{\mathrm{{cat}}}}}}{{K_{\mathrm{{M}}}}} = \frac{{K_{{\mathrm{{cat}}}}}}{{K_{\mathrm{{M}}}}}{\mathrm{{min}}} + \left( {\frac{{\frac{{K_{{\mathrm{{cat}}}}}}{{K_{\mathrm{{M}}}}}\max - \frac{{K_{{\mathrm{{cat}}}}}}{{K_{\mathrm{{M}}}}}{\mathrm{{min}}}}}{{1 + 10^{({\mathrm{{p}}}K_{{\mathrm{{a}}}} - {\mathrm{{pH}}}) \times {\mathrm{{HillSlope}}}}}}} \right)$$

### UPLC with procainamide labeled glycans

The enzymatic activity of wild type EnvSia156 and H134A mutant were tested by incubating 0.05, 0.15, 0.3, and 0.6 µg of enzyme with procainamide labeled 3’sialyllactose substrate in 50 mM sodium acetate pH 5.5, for 1 h at 37 °C. After incubation, 2.4 μL of sample were mixed with 17.6 µL acetonitrile for a 12:88 ratio. Sixteen microliters were injected into a Waters Acquity BEH glycan amide column (2.1 × 150 mm, 1.7 μm) on a Waters ACQUITY UPLC H-Class instrument (Waters Corporation, Milford, MA) equipped with a quaternary solvent manager and a fluorescence detector. Solvents used were A: 50 mM ammonium formate buffer pH 4.4 and B: 100% acetonitrile. The gradient used was 0–1.50 min, 12% solvent A; 1.5–35 min, 47% solvent A; 35–36.5 min, 70% solvent A; 36.5–42 min, 12% solvent A with a flow rate of 0.561 mL/min. Samples were kept at 5 °C prior to injection and separation was performed at 30 °C. The fluorescence detection wavelengths were *λ*_ex_ = 308 nm and *λ*_em_ = 359 nm with a data collection rate of 20 Hz. Data were analyzed with Empower 3 chromatography workstation software (Waters Corporation).

### SEC-MALLS

SEC-MALLS experiments were run in 20 mM HEPES 7.4, 300 mM NaCl buffer. The injected sample comprised 100 µL of EnvSia156 at 1.8 mg mL^−1^ in 20 mM HEPES pH 7.4, 100 mM NaCl, 1 mM DTT. Experiments were conducted on a system comprising a Superdex 200 10/30 GL (GE Healthcare) size exclusion chromatography column, a Wyatt HELEOS-II multi-angle light scattering detector and a Wyatt rEX refractive index detector linked to a Shimadzu HPLC system (SPD-20A UV detector, LC20-AD isocratic pump system, DGU-20A3 degasser, and SIL-20A autosampler). Work was conducted at room temperature (20 ± 2 °C). All solvents and buffers were 0.2 µm filtered before use and a further 0.1 µm filter was present in the flow path. Shimadzu LC Solutions software was used to control the HPLC and Astra V software for the HELEOS-II and rEX detectors. All data were analyzed using the Astra V software. Molecular masses were estimated using the Zimm fit method with degree 1. A value of 0.16 mL g^−1^ was used for protein refractive index increment (d*n*/d*c*), after calibration with a 2.5 mg mL^−1^ sample of BSA.

### Differential scanning fluorimetry

DSF experiments were performed in eight 0.2 mL polypropylene PCR tube strips (Agilent) with a final volume of 25 µL per tube. The sample mixture contained EnvSia156 (at 12.5, 25, 50, or 100 µM) and Sypro Orange dye (200 ng µL^−1^) in McIlvaine buffer at pH 5.5 and 7.5. Melting data were collected using the Agilent Stratagene Mx3005P rtPCR machine ramping from 25 to 95 °C at 30 s per degree. Data were analyzed using the JTSA server (http://paulsbond.co.uk/jtsa). Data points to the right of the highest fluorescence and to the left of the lowest fluorescence were discarded. The remaining points were fitted with a five parameter sigmoid equation (Sigmoid-5) model using the Levenberg–Marquardt algorithm.

### Cell culture

K562 cells (ATCC) were grown in RPMI 1640 medium (Corning, MT10040CV) + 10% heat-inactivated fetal bovine serum (heat inactivated) (Corning, 35016CV) + 1% penicillin/streptomycin (Fisher Scientific, sv30010), and tested negative for mycoplasma contamination by the MycoAlert PLUS mycoplasma test kit (Lonza, LT07-710).

### *Vibrio cholerae* NanH sialidase expression

The *V. cholerae* sialidase expressing plasmid pCVD364 expressed in *E*. coli C600 cells was a gift from Eric R. Vimr at the University of Illinois, Urbana-Champaign^67^. For expression, 6 mL of starter culture was grown for 8–12 h and used to inoculate 3 L of 2xYT media supplemented with 100 μg mL^−1^ carbenicillin at 37 °C for 12 h. Cells were pelleted by centrifugation at 4000 × *g* for 10 min. The pellet was resuspended in osmotic shock buffer (20% (w/vol) sucrose, 1 mM EDTA, 30 mM Tris–HCl, pH 8.0) and shaken gently for 10 min at room temperature. Cells were collected by centrifugation (9000 × *g* for 10 min), and resuspended in ice-cold water. After a 10 min incubation at 4 °C, the supernatant was obtained by centrifugation at 9000 × *g* for 10 min. The sample was concentrated using an Amicon ultrafiltration filter (MWCO = 50,000 Da), reconstituted in 20 mM M Tris–HCl buffer pH 7.6, run through a 0.2 μm syringe filter, and loaded onto a HitrapQ-HP anion-exchange column (17-1154-01; GE Healthcare Life Sciences) on an ÄKTA pure protein purification system at a flow rate of 3 mL/min. Buffers used were A: 20 mM Tris–HCl pH 7.6 and B: 20 mM Tris–HCl pH 7.6 + 1 M NaCl. The gradient used was 0–10 CV, hold at 0% B, 10–14 CV, linear gradient to 10% B, 14–26 CV, linear gradient to 30% B, 26–31 CV, step to 100% B. The majority of sialidase eluted around 22% B; these sialidase fractions were collected and pooled. Endotoxins were removed using a high-capacity endotoxin removal spin kit (88275; Thermo Fisher Scientific). Concentration was determined using the protein absorbance at 280 nm using the Nanodrop 2000 Spectrophotometer and the calculated molar extinction coefficient of 131780 M^−1^ cm^−1^.

### Lectin staining

K562 cells were resuspended in normal growth media and 300,000 cells/well were distributed into a V-bottom 96-well plate (Fisher Scientific, 0720096). Sialidases or PBS were added to respective wells to final concentrations of 4.25 μM (EnvSia156), 0.2 μM (*Vibrio cholerae* nanH sialidase), or equivalent volume PBS as a control. Cells were incubated with sialidase for 2 h at 37 °C, 5% CO_2_. Cells were then pelleted by centrifugation at 500 × *g* for 5 min. Supernatant was removed and replaced with 200 μL of biotinylated lectins at 10 μg mL^−1^ in cold PBS + 0.5% BSA (Sigma A9647-100G), lectins: biotinylated *Sambucus nigra* lectin (SNA) (Vector Labs, B-1305), or biotinylated *Maackia amurensis* lectin II (MAL II) (Vector Labs, B-1265). Cells were incubated with lectins for 30 min at 4 °C, followed by three washes (centrifugation at 500×*g* for 5 min, removal of supernatant, and resuspension in cold PBS + 0.5% BSA). After the final wash, cells were resuspended in 2.5 μg mL^−1^ streptavidin-Alexa Fluor 647 (Thermo Fisher Scientific, S21374) in cold PBS + 0.5% BSA and incubated at 4 °C for 15 min. Three more washes were performed and the cells were resuspended in PBS + 0.5% BSA containing 100 nM Sytox Green (Thermo Fisher Scientific, S7020) and analyzed on an LSR II flow cytometer (BD Biosciences). Cells were gated as shown in Supplementary Fig. 6; gating was performed using FlowJo v10.0 software to eliminate debris (FSC/SSC), analyze only single cells (FSC-A/FSC-H), and then select for live cells (Sytox green negative). Three independently performed experiments following this protocol were performed, all had >25,000 cells in the final gated population. The fold change in geometric mean fluorescence intensity (gMFI) over cells that were not lectin stained was plotted using GraphPad Prism v6 in Supplementary Fig. 7.

### Glycan array

Glycan array screening was performed by the Protein–Glycan Interaction Resource of the Consortium for Functional Glycomics, located at the National Center for Functional Glycomics (NCFG), Beth Israel Deaconess Medical Center, Harvard Medical School. The binding of an inactive variant (D14A mutant) of EnvSia156 was tested using the CFG-printed array version 5.4, which consists of 585 glycans in replicates of 6. The experiment was performed as described in https://ncfg.hms.harvard.edu/protocols/glycan-binding-assay-fusion-or-epitope-tagged-protein, using 5, 50, and 200 μg mL^−1^ of EnvSia156 and 5 μg mL^−1^ of Alexa-Fluor-488-labeled anti-His antibody for detection of bound sample.

### Reporting summary

Further information on research design is available in the Nature Research Reporting Summary linked to this article.

## Supplementary information

Supplementary Information

Reporting Summary

Source Data

## Data Availability

Coordinates and structure factors have been deposited in the Protein Data Bank under accession codes PDB 6RZD [https://www.rcsb.org/structure/6RZD] (unliganded structure), PDB 6S00 [https://www.rcsb.org/structure/6S00] (Neu5Ac complex), PDB 6S04 [https://www.rcsb.org/structure/6S04] (Neu5Gc complex), PDB 6S0E [https://www.rcsb.org/structure/6S0E] (DANA complex) and PDB 6S0F [https://www.rcsb.org/structure/6S0F] (KDN complex). The source data underlying Fig. 1 and Supplementary Figs. 1, 4a, 7b and 8 are provided as a Source Data file. All further data supporting the findings of this study are available from the corresponding authors, upon reasonable request.
