# Supplementary material for: Inverting family GH156 sialidases define an unusual catalytic motif for glycosidase action
Source: Nat Commun. 2019 Oct 23;10:4816. doi: 10.1038/s41467-019-12684-7 (PMC6811678; doi:10.1038/s41467-019-12684-7)
Supplement: Supplementary file 1 — Supplementary Information [file 41467_2019_12684_MOESM1_ESM.pdf]

SUPPLEMENTARY INFORMATION

Inverting family GH156 sialidases define an unusual catalytic motif for glycosidase action

Bule *et al*.

SUPPLEMENTARY FIGURES

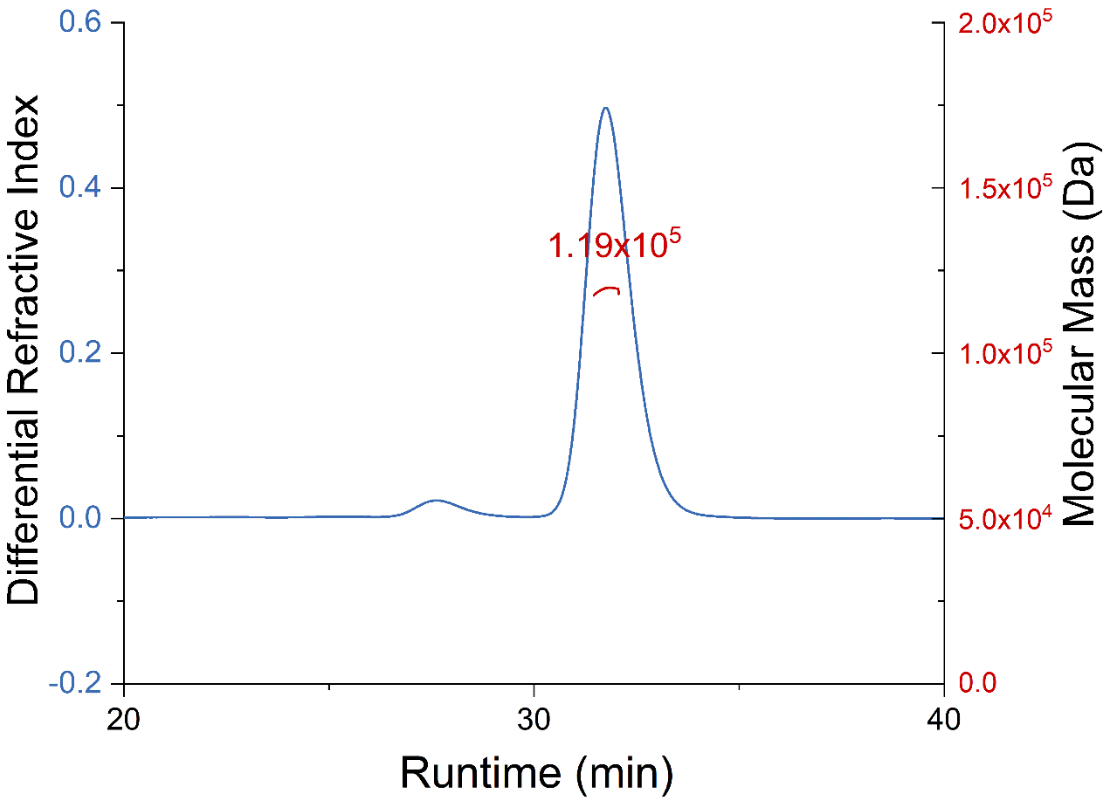

Supplementary Figure 1. Analysis of the solution oligomeric state of EnvSia156 by SEC-MALLS. SEC-MALLS trace showing a large peak for purified EnvSia156, with an estimated molecular mass of 119 kDa, consistent with a dimeric assembly. A second discrete peak can be observed which may correspond to a tetrameric assembly (dimer of a dimer). Source data are provided as a Source Data file.

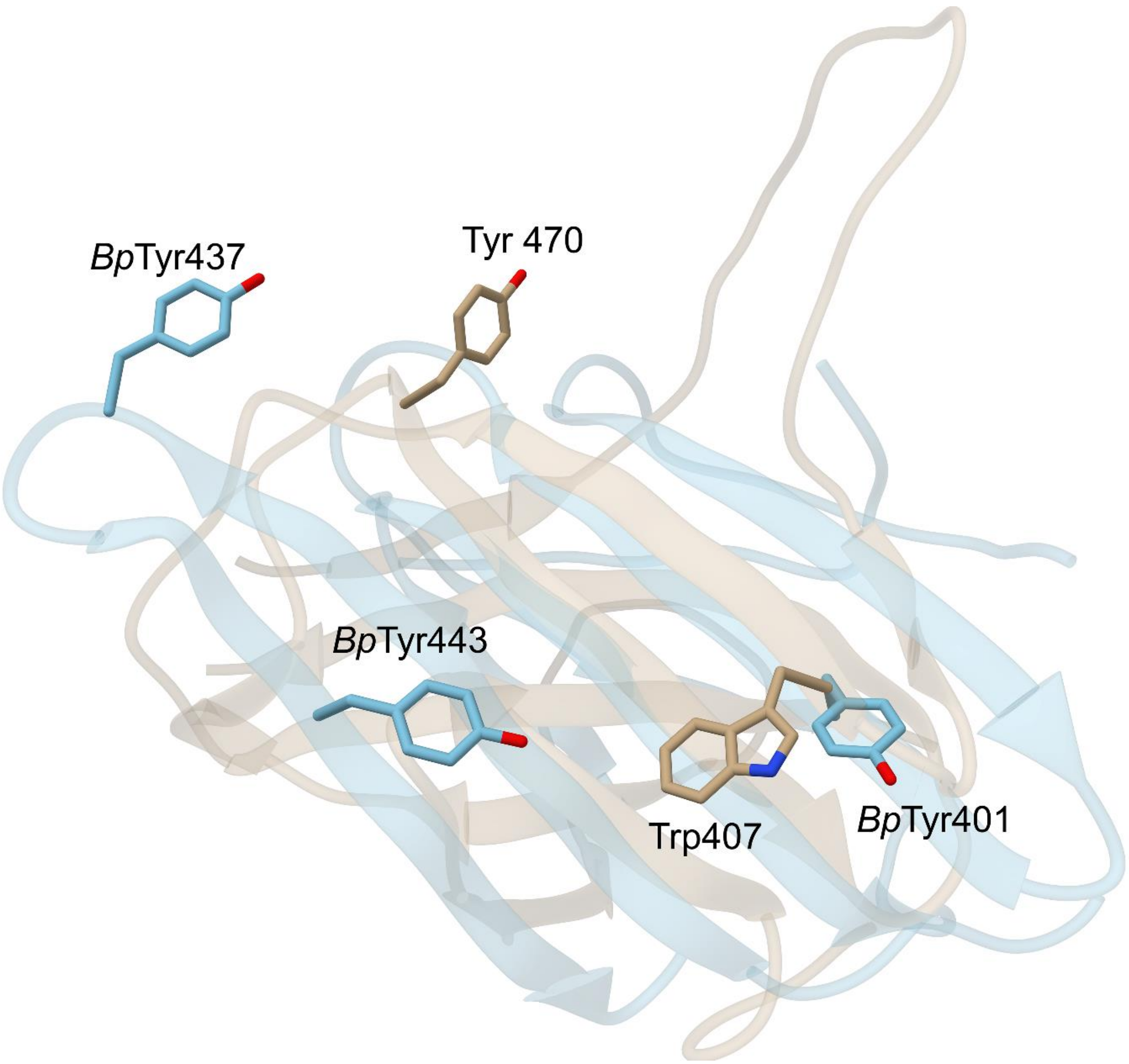

Supplementary Figure 2. Structural homology between EnvSia156 β-barrel domain and a family 4 CBM. Superposition of the C-terminal β-barrel domain of EnvSia156 (brown) with a CBM4 from a porphiranase produced by *Bacteroides plebeius* (light blue). The structures were aligned using the Needleman-Wunsch algorithm and BLOSSUM 62 matrix of the matchmaker function in UCSF Chimera. Carbohydrate interacting surface aromatic residues from *Bt*CBM4 are displayed in stick representation, as well as the two surface aromatics present on EnvSia156 matching side.

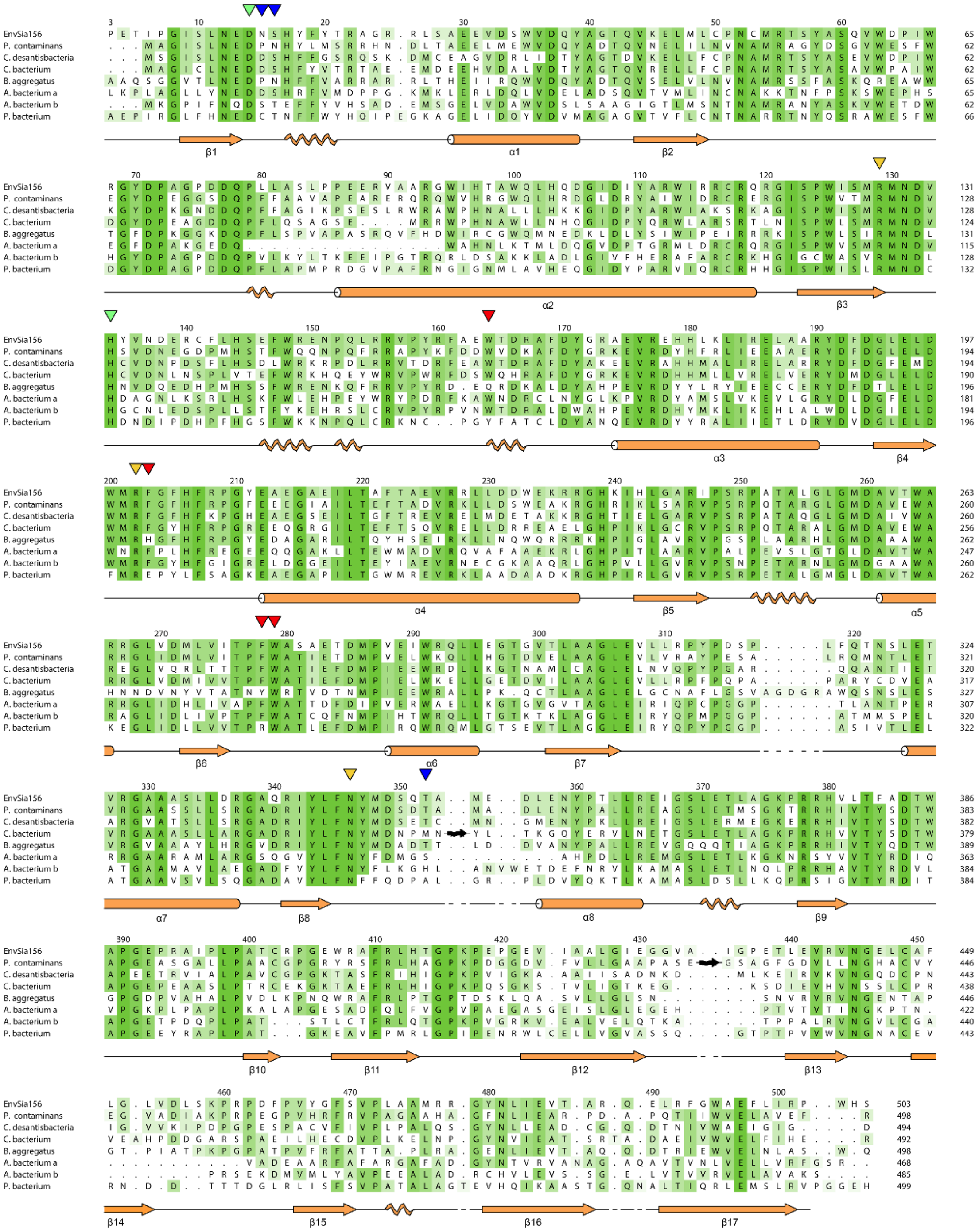

Supplementary Figure 3. Alignment with closest homologs. Alignment of EnvSia156 with the seven closest sequence homologs found with the BlastP tool. All proteins belong to bacteria, namely *Paenibacillus contaminans*, *Candidatus desantisbacteria*, *Chloroflexi bacterium*, *Bryobacter aggregatus*, *Armatimonadetes bacterium* (2 sequences), *Planctomycetes bacterium*. A cartoon representation of EnvSia156 secondary structure is displayed in orange, below the alignment. The sequences were aligned using the Clustal Omega tool and further processed with ALINE. Colouring according to similarity was implemented with ALINE: dark green, identical residues; green to white, lowering color-ramped scale of conservation. Green arrows point to the EnvSia156 putative catalytic pair, yellow arrows to the carboxylate coordinating triad, red arrows to the residues defining the predicted +1 subsite and the blue arrows to the remaining residues establishing contacts with Neu5Ac as seen in the EnvSia156Neu5Ac complex structure.

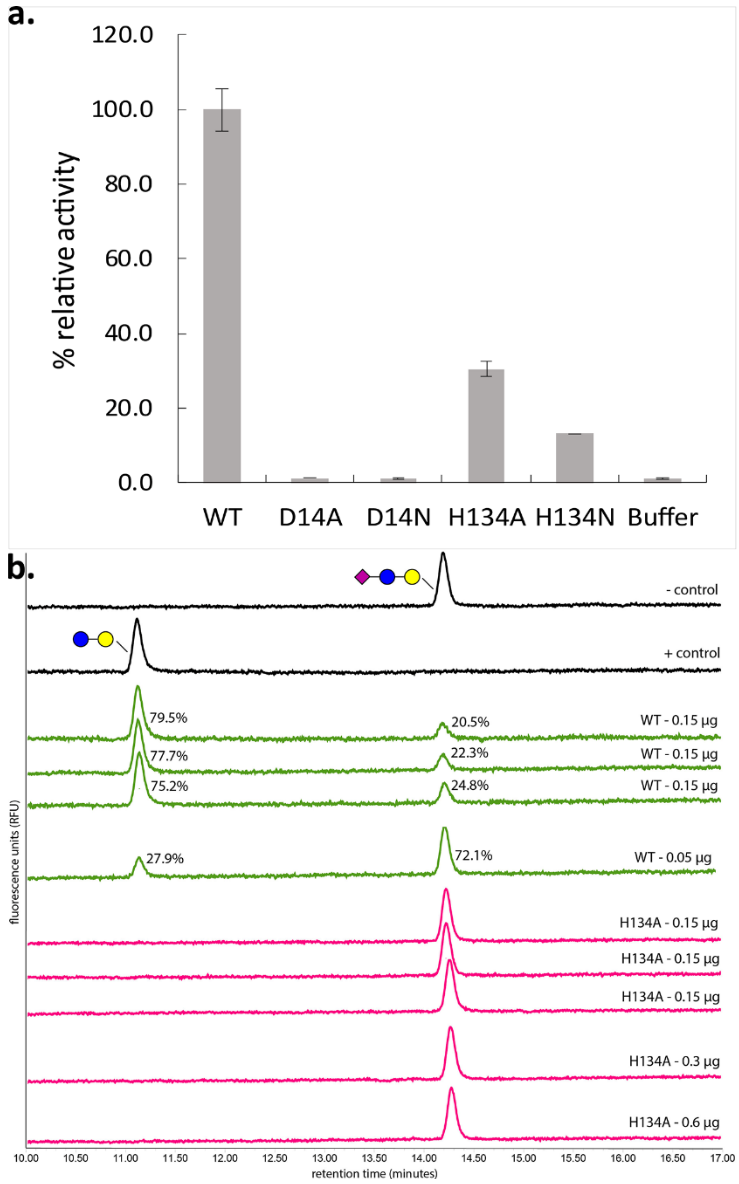

Supplementary Figure 4. Activity of EnvSia156 mutants (a.) Relative activity of EnvSia156 mutants determined by hydrolysis of 4MU-α-Neu5Ac substrate. Error bars indicate the standard deviation of three replicates. **(b)** Relative activity of EnvSia156 H134A mutant determined by digestion of procrainamide labelled α-2,3-sialyl-lactose with detection of both substrate and product relative amounts by UPLC. Experiments with WT – 0.15 µg and H134A 0.15 µg were performed in triplicate. Source data are provided as a Source Data file.

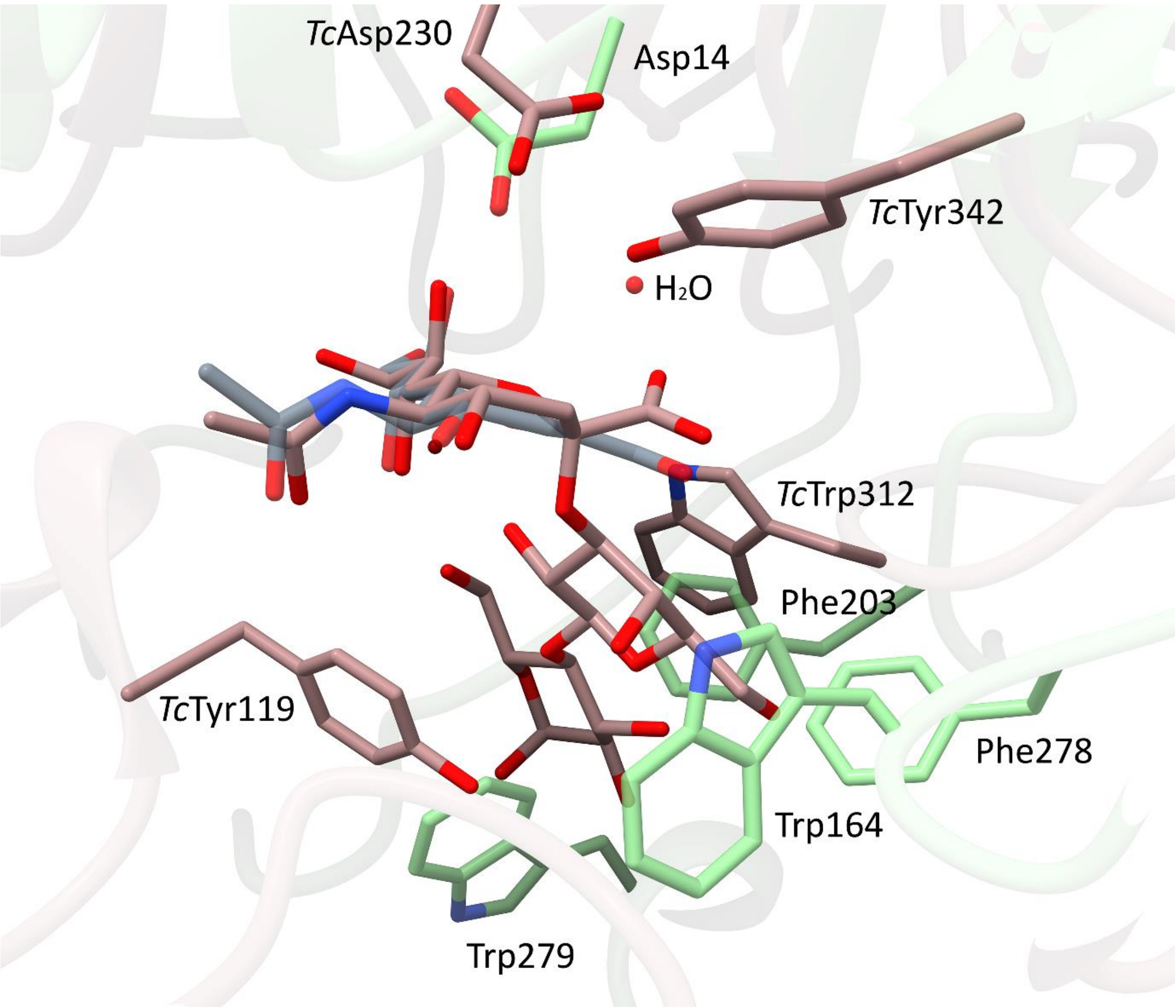

Supplementary Figure 5. EnvSia156 vs *T. cruzi* sialidase active site conformation. Superposition of the EnvSia156DANA complex with a *T. cruzi* sialidase complex with sialyl-lactose. EnvSia156 is represented with light green ribbons, *T. cruzi* trans-sialidase is represented with pink ribbons, DANA is in transparent grey stick representation and sialyl-lactose is in a pink stick representation.

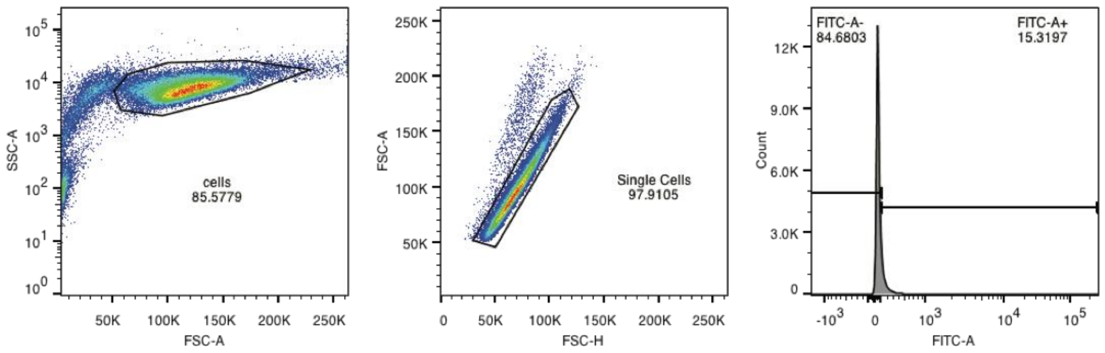

Supplementary Figure 6. Gating strategy for flow cytometry of sialidase-treated K562 cells. Gating was performed around the K562 population in the SSC-A/FSC-A channel, followed by gating on single cells (FSC-H/FSC-A). Live singlet K562 cells were then isolated by gating for Sytox green-negative cells in the FITC laser channel. For unstained or secondary only stained cells, >95% of isolated single cells were live, for lectin stained cells 50-90% of singlet cells were live due to cytotoxic effects of lectin binding during staining.

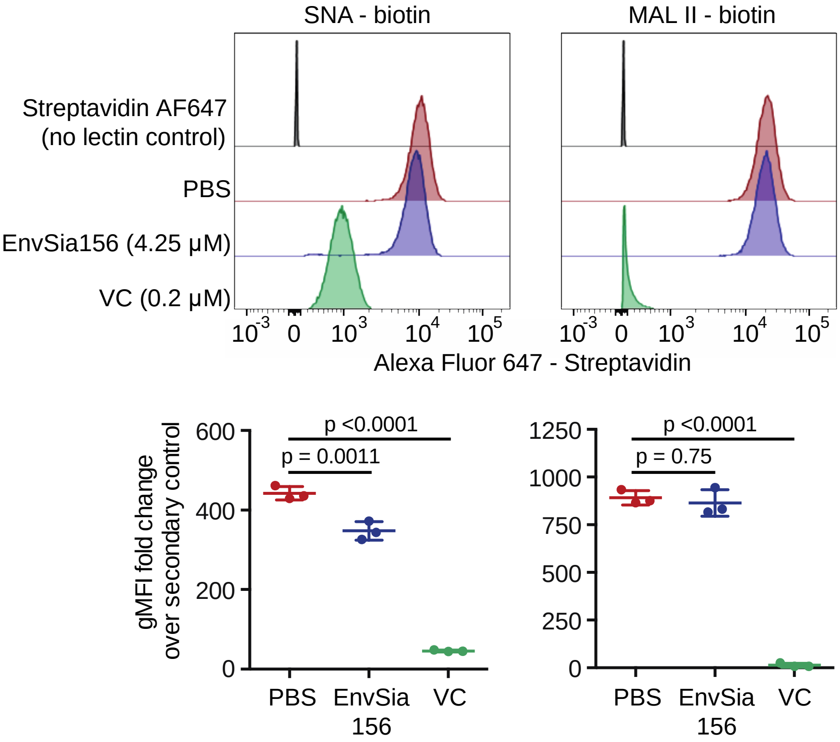

Supplementary Figure 7. Treating cells with high concentrations of EnvSia156 slightly reduced SNA lectin binding, but make no significant difference in MAL II lectin binding. Above: representative histograms of n=3 independently performed experiments where K562 cells treated with EnvSia156 (4.25 μM), *Vibrio cholerae* sialidase (VC) (200 nM), or PBS were stained with biotinylated *Sambucus nigra* lectin (SNA, left) or biotinylated *Maackia amurensis* lectin II (MAL II, right). Streptavidin-Alexa Fluor 647 fluorescence intensity is shown on the biexponential scale on the x axis, and the y-axis is normalized to mode, all histograms display a population of >25,000 cells. Below: the geometric mean fluorescence intensity (gMFI) fold change in EnvSia156, VC, or PBS over control cells treated only with streptavidin-Alexa Fluor 647. Data were analyzed using an ordinary one-way ANOVA, and multiplicity-adjusted post-hoc p-values are reported from a Tukey multiple comparison test. Source data are provided as a Source Data file.

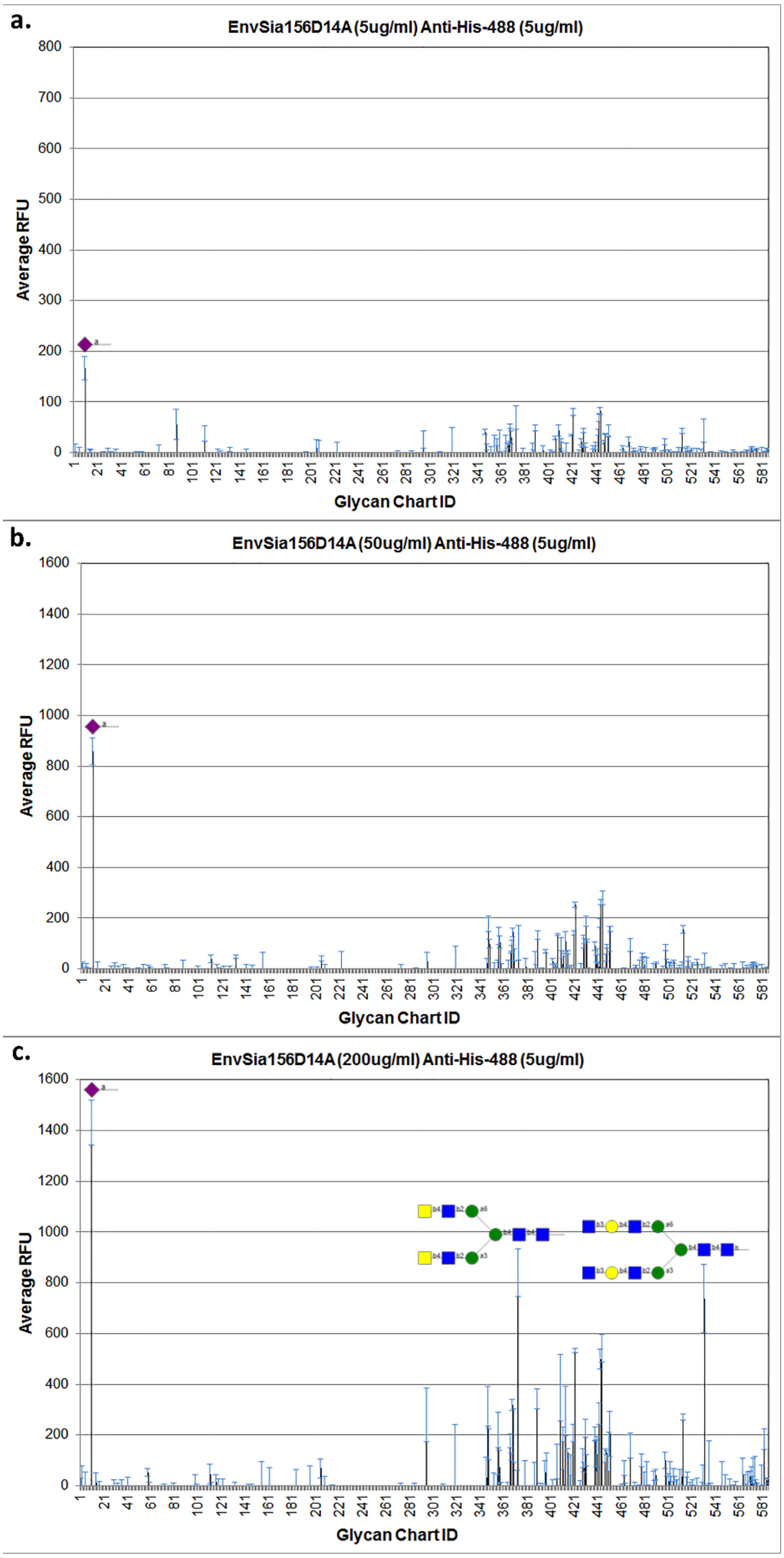

Supplementary Figure 8. Glycan Microarray shows binding to Neu5Ac. Bar plots showing Glycan Chart ID (Supplementary table 3) vs. Average RFU with standard error of the mean plotted in the error bars. The experiment was performed using 5 µg/mL (a.), 50 µg/mL (b.) and 200 µg/mL (c.). The highest and lowest point from each set of 6 replicates has been removed so the average is of 4 values rather than 6. This eliminates some of the false hits that contain a single very high or low point. Source data are provided as a Source Data file.

SUPPLEMENTARTY TABLES

	SeMet Apo			Apo	Neu5Ac	Neu5Gc	DANA	KDN
**Data collection**	peak	edge	remote					
Space group	$P2_1$			$P2_1$	$P2_1$	$P2_1$	$P2_1$	$P2_1$
Cell dimensions								
a, *b*, *c* (Å)	64.33, 79.37, 112.51			64.11, 79.27, 112.21	64.28, 79.03, 112.93	65.05, 79.55, 113.52	63.43, 78.75, 112.80	63.66, 78.92, 112.90
α, *β*, *γ* (°)	90.00, 94.76, 90.00			90.00, 94.72, 90.00	90.00,94.88, 90.00	90.00, 94.95 90.00	90.00, 94.94, 90.00	90.00, 94.94, 90.00
Resolution (Å)	1.78 – 64.15 (1.78 – 1.83)	1.72 – 64.22 (1.72 – 1.76)	1.88 – 64.82 (1.88 – 1.93)	64.67 – 2.00 (2.04 – 2.00)	49.75 – 2.00 (2.04 – 2.00)	65.07 – 2.00 (2.04 – 2.00)	78.76 – 1.90 (1.9 – 1.93)	112.58 – 2.00 (2.04 – 2.00)
R_{merge}	0.191 (1.381)	0.156 (1.542)	0.213 (1.419)	0.198 (0.707))	0.292 (1.927)	0.236 (0.635)	0.170 (0.553)	0.162 (0.576)
R_{pim}	0.081 (0.598)	0.067 (0.727)	0.091 (0.611)	0.094 (0.377)	0.071 (0.466)	0.126 (0.342)	0.133 (0.433)	0.127 (0.457)
CC(1/2)	0.991 (0.576)	0.995 (0.478)	0.989 (0.664)	0.985 (0.593)	0.993 (0.710)	0.963 (0.739)	0.901 (0.756)	0.956 (0.848)
I / σI	6.3 (1.1)	7.9 (1.2)	5.6 (1.1)	5.8 (1.8)	10.3 (1.6)	3.7 (2.1)	5.0 (2.5)	5.4 (2.4)
Completeness (%)	100 (100)	100 (100)	100 (100)	100 (99.7)	100 (100)	100 (100)	99.9 (99.9)	99.9 (100)
Redundancy	6.4 (6.3)	6.3 (5.4)	6.5 (6.4)	5.4 (5.6)	18.0 (17.7)	4.1 (4.2)	4.0 (3.8)	4.0 (4.2)
Anomalous Completeness	99.7 (99.5)	99.7 (99.3)	66.7 (99.6)					
Anomalous Redundancy	3.3 (3.1)	3.2 (2.7)	3.3 (3.2)					
**Refinement**								
No. reflections				494271 (49865)	1371172 (78658)	324149 (32573)	347962 (34186)	303814 (31068)
R_{work} / R_{free}	0.23 / 0.27			0.23 / 0.29	0.21 / 0.25	0.23 / 0.28	0.25 / 0.31)	0.22 / 0.27
No. atoms								
Protein				8048	8033	8038	8034	8058
Ligand/ion				106	121	84	128	125
Water				420	477	550	490	487
B-factors (Å^2)								
Protein				24.70	24.52	18.17	22.33	22.80
Ligand/ion				35.88	30.53	21.83	26.63	31.64
Water				24.02	26.95	20.12	22.93	27.37
R.m.s. deviations								
Bond lengths (Å)				0.017	0.016	0.017	0.017	0.017
Bond angles (°)				2.10	2.01	2.09	2.11	2.12
Ramachandran plot residues								
In most favourable regions(%)				96.04	96.85	96.25	95.63	96.76
In allowed regions (%)				3.56	2.95	3.45	4.07	2.84
**PDB code**				**6RZD**	**6S00**	**6S04**	**6S0E**	**6S0F**

Supplementary table 1. Data collection and refinement statistics for all EnvSia156 structures. Data collection statistics were obtained from the aimless log while refinement statistics were calculated using Phoenix Table 1 utility. Statistics for the highest resolution shell are between parentheses.

Chart ID	Glycan structure
1	Gala-Sp8
2	Glca-Sp8
3	Mana-Sp8
4	GalNAca-Sp8
5	GalNAca-Sp15
6	Fuca-Sp8
7	Fuca-Sp9
8	Rhaa-Sp8
9	Neu5Aca-Sp8
10	Neu5Aca-Sp11
11	Neu5Acb-Sp8
12	Galb-Sp8
13	Glcb-Sp8
14	Manb-Sp8
15	GalNAcb-Sp8
16	GlcNAcb-Sp0
17	GlcNAcb-Sp8
18	GlcN(Gc)b-Sp8
19	Galb1-4GlcNAcb1-6(Galb1-4GlcNAcb1-3)GalNAca-Sp8
20	Galb1-4GlcNAcb1-6(Galb1-4GlcNAcb1-3)GalNAc-Sp14
21	GlcNAcb1-6(GlcNAcb1-4)(GlcNAcb1-3)GlcNAc-Sp8
22	6S(3S)Galb1-4(6S)GlcNAcb-Sp0
23	6S(3S)Galb1-4GlcNAcb-Sp0
24	(3S)Galb1-4(Fuca1-3)(6S)Glc-Sp0
25	(3S)Galb1-4Glcb-Sp8
26	(3S)Galb1-4(6S)Glcb-Sp0
27	(3S)Galb1-4(6S)Glcb-Sp8
28	(3S)Galb1-3(Fuca1-4)GlcNAcb-Sp8
29	(3S)Galb1-3GalNAca-Sp8
30	(3S)Galb1-3GlcNAcb-Sp0
31	(3S)Galb1-3GlcNAcb-Sp8
32	(3S)Galb1-4(Fuca1-3)GlcNAc-Sp0
33	(3S)Galb1-4(Fuca1-3)GlcNAc-Sp8
34	(3S)Galb1-4(6S)GlcNAcb-Sp0
35	(3S)Galb1-4(6S)GlcNAcb-Sp8
36	(3S)Galb1-4GlcNAcb-Sp0
37	(3S)Galb1-4GlcNAcb-Sp8
38	(3S)Galb-Sp8
39	(6S)(4S)Galb1-4GlcNAcb-Sp0
40	(4S)Galb1-4GlcNAcb-Sp8
41	(6P)Mana-Sp8
42	(6S)Galb1-4Glcb-Sp0
43	(6S)Galb1-4Glcb-Sp8
44	(6S)Galb1-4GlcNAcb-Sp8
45	(6S)Galb1-4(6S)Glcb-Sp8
46	Neu5Aca2-3(6S)Galb1-4GlcNAcb-Sp8
47	(6S)GlcNAcb-Sp8
48	Neu5,9Ac$_2$a-Sp8
49	Neu5,9Ac2a2-6Galb1-4GlcNAcb-Sp8
50	Mana1-6(Mana1-3)Manb1-4GlcNAcb1-4GlcNAcb-Sp12
51	Mana1-6(Mana1-3)Manb1-4GlcNAcb1-4GlcNAcb-Sp13
52	GlcNAcb1-2Mana1-6(GlcNAcb1-2Mana1-3)Manb1-4GlcNAcb1-4GlcNAcb-Sp12
53	GlcNAcb1-2Mana1-6(GlcNAcb1-2Mana1-3)Manb1-4GlcNAcb1-4GlcNAcb-Sp13
54	Galb1-4GlcNAcb1-2Mana1-6(Galb1-4GlcNAcb1-2Mana1-3)Manb1-4GlcNAcb1-4GlcNAcb-Sp12
55	Neu5Aca2-6Galb1-4GlcNAcb1-2Mana1-6(Neu5Aca2-6Galb1-4GlcNAcb1-2Mana1-3)Manb1-4GlcNAcb1-4GlcNAcb-Sp12
56	Neu5Aca2-6Galb1-4GlcNAcb1-2Mana1-6(Neu5Aca2-6Galb1-4GlcNAcb1-2Man-a1-3)Manb1-4GlcNAcb1-4GlcNAcb-Sp21
57	Neu5Aca2-6Galb1-4GlcNAcb1-2Mana1-6(Neu5Aca2-6Galb1-4GlcNAcb1-2Mana1-3)Manb1-4GlcNAcb1-4GlcNAcb-Sp24
58	Fuca1-2Galb1-3GalNAcb1-3Gala-Sp9
59	Fuca1-2Galb1-3GalNAcb1-3Gala1-4Galb1-4Glcb-Sp9
60	Fuca1-2Galb1-3(Fuca1-4)GlcNAcb-Sp8
61	Fuca1-2Galb1-3GalNAca-Sp8
62	Fuca1-2Galb1-3GalNAca-Sp14
63	Fuca1-2Galb1-3GalNAcb1-4(Neu5Aca2-3)Galb1-4Glcb-Sp0
64	Fuca1-2Galb1-3GalNAcb1-4(Neu5Aca2-3)Galb1-4Glcb-Sp9
65	Fuca1-2Galb1-3GlcNAcb1-3Galb1-4Glcb-Sp8
66	Fuca1-2Galb1-3GlcNAcb1-3Galb1-4Glcb-Sp10
67	Fuca1-2Galb1-3GlcNAcb-Sp0

68	Fuca1-2Galb1-3GlcNAcb-Sp8
69	Fuca1-2Galb1-4(Fuca1-3)GlcNAcb1-3Galb1-4(Fuca1-3)GlcNAcb-Sp0
70	Fuca1-2Galb1-4(Fuca1-3)GlcNAcb1-3Galb1-4(Fuca1-3)GlcNAcb1-3Galb1-4(Fuca1-3)GlcNAcb-Sp0
71	Fuca1-2Galb1-4(Fuca1-3)GlcNAcb-Sp0
72	Fuca1-2Galb1-4(Fuca1-3)GlcNAcb-Sp8
73	Fuca1-2Galb1-4GlcNAcb1-3Galb1-4GlcNAcb-Sp0
74	Fuca1-2Galb1-4GlcNAcb1-3Galb1-4GlcNAcb1-3Galb1-4GlcNAcb-Sp0
75	Fuca1-2Galb1-4GlcNAcb-Sp0
76	Fuca1-2Galb1-4GlcNAcb-Sp8
77	Fuca1-2Galb1-4Glcb-Sp0
78	Fuca1-2Galb-Sp8
79	Fuca1-3GlcNAcb-Sp8
80	Fuca1-4GlcNAcb-Sp8
81	Fucb1-3GlcNAcb-Sp8
82	GalNAca1-3(Fuca1-2)Galb1-3GlcNAcb-Sp0
83	GalNAca1-3(Fuca1-2)Galb1-4(Fuca1-3)GlcNAcb-Sp0
84	(3S)Galb1-4(Fuca1-3)Glcb-Sp0
85	GalNAca1-3(Fuca1-2)Galb1-4GlcNAcb-Sp0
86	GalNAca1-3(Fuca1-2)Galb1-4GlcNAcb-Sp8
87	GalNAca1-3(Fuca1-2)Galb1-4Glcb-Sp0
88	GlcNAcb1-3Galb1-3GalNAca-Sp8
89	GalNAca1-3(Fuca1-2)Galb-Sp8
90	GalNAca1-3(Fuca1-2)Galb-Sp18
91	GalNAca1-3GalNAcb-Sp8
92	GalNAca1-3Galb-Sp8
93	GalNAca1-4(Fuca1-2)Galb1-4GlcNAcb-Sp8
94	GalNAcb1-3GalNAca-Sp8
95	GalNAcb1-3(Fuca1-2)Galb-Sp8
96	GalNAcb1-3Gala1-4Galb1-4GlcNAcb-Sp0
97	GalNAcb1-4(Fuca1-3)GlcNAcb-Sp0
98	GalNAcb1-4GlcNAcb-Sp0
99	GalNAcb1-4GlcNAcb-Sp8
100	Gala1-2Galb-Sp8
101	Gala1-3(Fuca1-2)Galb1-3GlcNAcb-Sp0
102	Gala1-3(Fuca1-2)Galb1-3GlcNAcb-Sp8
103	Gala1-3(Fuca1-2)Galb1-4(Fuca1-3)GlcNAcb-Sp0
104	Gala1-3(Fuca1-2)Galb1-4(Fuca1-3)GlcNAcb-Sp8
105	Gala1-3(Fuca1-2)Galb1-4GlcNAc-Sp0
106	Gala1-3(Fuca1-2)Galb1-4Glcb-Sp0
107	Gala1-3(Fuca1-2)Galb-Sp8
108	Gala1-3(Fuca1-2)Galb-Sp18
109	Gala1-4(Gala1-3)Galb1-4GlcNAcb-Sp8
110	Gala1-3GalNAca-Sp8
111	Gala1-3GalNAca-Sp16
112	Gala1-3GalNAcb-Sp8
113	Gala1-3Galb1-4(Fuca1-3)GlcNAcb-Sp8
114	Gala1-3Galb1-3GlcNAcb-Sp0
115	Gala1-3Galb1-4GlcNAcb-Sp8
116	Gala1-3Galb1-4Glcb-Sp0
117	Gala1-3Galb1-4Glc-Sp10
118	Gala1-3Galb-Sp8
119	Gala1-4(Fuca1-2)Galb1-4GlcNAcb-Sp8
120	Gala1-4Galb1-4GlcNAcb-Sp0
121	Gala1-4Galb1-4GlcNAcb-Sp8
122	Gala1-4Galb1-4Glcb-Sp0
123	Gala1-4GlcNAcb-Sp8
124	Gala1-6Glcb-Sp8
125	Galb1-2Galb-Sp8
126	Galb1-3(Fuca1-4)GlcNAcb1-3Galb1-4(Fuca1-3)GlcNAcb-Sp0
127	Galb1-3GlcNAcb1-3Galb1-4(Fuca1-3)GlcNAcb-Sp0
128	Galb1-3(Fuca1-4)GlcNAc-Sp0
129	Galb1-3(Fuca1-4)GlcNAc-Sp8
130	Fuca1-4(Galb1-3)GlcNAcb-Sp8
131	Galb1-4GlcNAcb1-6GalNAca-Sp8
132	Galb1-4GlcNAcb1-6GalNAc-Sp14
133	GlcNAcb1-6(Galb1-3)GalNAca-Sp8
134	GlcNAcb1-6(Galb1-3)GalNAca-Sp14
135	Neu5Aca2-6(Galb1-3)GalNAca-Sp8
136	Neu5Aca2-6(Galb1-3)GalNAca-Sp14
137	Neu5Acb2-6(Galb1-3)GalNAca-Sp8
138	Neu5Aca2-6(Galb1-3)GlcNAcb1-4Galb1-4Glcb-Sp10

139	Galb1-3GalNAca-Sp8
140	Galb1-3GalNAca-Sp14
141	Galb1-3GalNAca-Sp16
142	Galb1-3GalNAcb-Sp8
143	Galb1-3GalNAcb1-3Gala1-4Galb1-4Glcb-Sp0
144	Galb1-3GalNAcb1-4(Neu5Aca2-3)Galb1-4Glcb-Sp0
145	Galb1-3GalNAcb1-4Galb1-4Glcb-Sp8
146	Galb1-3Galb-Sp8
147	Galb1-3GlcNAcb1-3Galb1-4GlcNAcb-Sp0
148	Galb1-3GlcNAcb1-3Galb1-4Glcb-Sp10
149	Galb1-3GlcNAcb-Sp0
150	Galb1-3GlcNAcb-Sp8
151	Galb1-4(Fuca1-3)GlcNAcb-Sp0
152	Galb1-4(Fuca1-3)GlcNAcb-Sp8
153	Galb1-4(Fuca1-3)GlcNAcb1-3Galb1-4(Fuca1-3)GlcNAcb-Sp0
154	Galb1-4(Fuca1-3)GlcNAcb1-3Galb1-4(Fuca1-3)GlcNAcb1-3Galb1-4(Fuca1-3)GlcNAcb-Sp0
155	Galb1-4(6S)Glcb-Sp0
156	Galb1-4(6S)Glcb-Sp8
157	Galb1-4GalNAca1-3(Fuca1-2)Galb1-4GlcNAcb-Sp8
158	Galb1-4GalNAcb1-3(Fuca1-2)Galb1-4GlcNAcb-Sp8
159	Galb1-4GlcNAcb1-3GalNAca-Sp8
160	Galb1-4GlcNAcb1-3GalNAc-Sp14
161	Galb1-4GlcNAcb1-3Galb1-4(Fuca1-3)GlcNAcb1-3Galb1-4(Fuca1-3)GlcNAcb-Sp0
162	Galb1-4GlcNAcb1-3Galb1-4GlcNAcb1-3Galb1-4GlcNAcb-Sp0
163	Galb1-4GlcNAcb1-3Galb1-4GlcNAcb-Sp0
164	Galb1-4GlcNAcb1-3Galb1-4Glcb-Sp0
165	Galb1-4GlcNAcb1-3Galb1-4Glcb-Sp8
166	Galb1-4GlcNAcb1-6(Galb1-3)GalNAca-Sp8
167	Galb1-4GlcNAcb1-6(Galb1-3)GalNAc-Sp14
168	Galb1-4GlcNAcb-Sp0
169	Galb1-4GlcNAcb-Sp8
170	Galb1-4GlcNAcb-Sp23
171	Galb1-4Glcb-Sp0
172	Galb1-4Glcb-Sp8
173	GlcNAca1-3Galb1-4GlcNAcb-Sp8
174	GlcNAca1-6Galb1-4GlcNAcb-Sp8
175	GlcNAcb1-2Galb1-3GalNAca-Sp8
176	GlcNAcb1-6(GlcNAcb1-3)GalNAca-Sp8
177	GlcNAcb1-6(GlcNAcb1-3)GalNAca-Sp14
178	GlcNAcb1-6(GlcNAcb1-3)Galb1-4GlcNAcb-Sp8
179	GlcNAcb1-3GalNAca-Sp8
180	GlcNAcb1-3GalNAca-Sp14
181	GlcNAcb1-3Galb-Sp8
182	GlcNAcb1-3Galb1-4GlcNAcb-Sp0
183	GlcNAcb1-3Galb1-4GlcNAcb-Sp8
184	GlcNAcb1-3Galb1-4GlcNAcb1-3Galb1-4GlcNAcb-Sp0
185	GlcNAcb1-3Galb1-4Glcb-Sp0
186	GlcNAcb1-4-MDPLys
187	GlcNAcb1-6(GlcNAcb1-4)GalNAca-Sp8
188	GlcNAcb1-4Galb1-4GlcNAcb-Sp8
189	GlcNAcb1-4GlcNAcb1-4GlcNAcb1-4GlcNAcb1-4GlcNAcb1-4GlcNAcb1-Sp8
190	GlcNAcb1-4GlcNAcb1-4GlcNAcb1-4GlcNAcb1-4GlcNAcb1-Sp8
191	GlcNAcb1-4GlcNAcb1-4GlcNAcb-Sp8
192	GlcNAcb1-6GalNAca-Sp8
193	GlcNAcb1-6GalNAca-Sp14
194	GlcNAcb1-6Galb1-4GlcNAcb-Sp8
195	Glca1-4Glcb-Sp8
196	Glca1-4Glca-Sp8
197	Glca1-6Glca1-6Glcb-Sp8
198	Glcb1-4Glcb-Sp8
199	Glcb1-6Glcb-Sp8
200	G-ol-Sp8
201	GlcAa-Sp8
202	GlcAb-Sp8
203	GlcAb1-3Galb-Sp8
204	GlcAb1-6Galb-Sp8
205	KDNa2-3Galb1-3GlcNAcb-Sp0
206	KDNa2-3Galb1-4GlcNAcb-Sp0
207	Mana1-2Mana1-2Mana1-3Mana-Sp9
208	Mana1-2Mana1-6(Mana1-2Mana1-3)Mana-Sp9
209	Mana1-2Mana1-3Mana-Sp9

210	Mana1-2Mana1-6(Mana1-2Mana1-3)Mana1-6(Mana1-2Mana1-2Mana1-3)Manb1-4GlcNAcb1-4GlcNAcb-Sp12
211	Mana1-6(Mana1-3)Mana-Sp9
212	Mana1-2Mana1-2Mana1-6(Mana1-3)Mana-Sp9
213	Mana1-6(Mana1-3)Mana1-6(Mana1-2Mana1-3)Manb1-4GlcNAcb1-4GlcNAcb-Sp12
214	Mana1-6(Mana1-3)Mana1-6(Mana1-3)Manb1-4GlcNAcb1-4GlcNAcb-Sp12
215	Manb1-4GlcNAcb-Sp0
216	Neu5Aca2-3Galb1-4GlcNAcb1-3Galb1-4(Fuca1-3)GlcNAcb-Sp0
217	(3S)Galb1-4(Fuca1-3)(6S)GlcNAcb-Sp8
218	Fuca1-2(6S)Galb1-4GlcNAcb-Sp0
219	Fuca1-2Galb1-4(6S)GlcNAcb-Sp8
220	Fuca1-2(6S)Galb1-4(6S)Glcb-Sp0
221	Neu5Aca2-3Galb1-3GalNAca-Sp8
222	Neu5Aca2-3Galb1-3GalNAca-Sp14
223	GalNAcb1-4(Neu5Aca2-8Neu5Aca2-8Neu5Aca2-8Neu5Aca2-3)Galb1-4Glcb-Sp0
224	GalNAcb1-4(Neu5Aca2-8Neu5Aca2-8Neu5Aca2-3)Galb1-4Glcb-Sp0
225	Neu5Aca2-8Neu5Aca2-8Neu5Aca2-3Galb1-4Glcb-Sp0
226	GalNAcb1-4(Neu5Aca2-8Neu5Aca2-3)Galb1-4Glcb-Sp0
227	Neu5Aca2-8Neu5Aca2-8Neu5Aca-Sp8
228	GalNAcb1-4(Neu5Aca2-3)Galb1-4GlcNAcb-Sp0
229	GalNAcb1-4(Neu5Aca2-3)Galb1-4GlcNAcb-Sp8
230	GalNAcb1-4(Neu5Aca2-3)Galb1-4Glcb-Sp0
231	Neu5Aca2-3Galb1-3GalNAcb1-4(Neu5Aca2-3)Galb1-4Glcb-Sp0
232	Neu5Aca2-6(Neu5Aca2-3)GalNAca-Sp8
233	Neu5Aca2-3GalNAca-Sp8
234	Neu5Aca2-3GalNAcb1-4GlcNAcb-Sp0
235	Neu5Aca2-3Galb1-3(6S)GlcNAc-Sp8
236	Neu5Aca2-3Galb1-3(Fuca1-4)GlcNAcb-Sp8
237	Neu5Aca2-3Galb1-3(Fuca1-4)GlcNAcb1-3Galb1-4(Fuca1-3)GlcNAcb-Sp0
238	Neu5Aca2-3Galb1-4(Neu5Aca2-3Galb1-3)GlcNAcb-Sp8
239	Neu5Aca2-3Galb1-3(6S)GalNAca-Sp8
240	Neu5Aca2-6(Neu5Aca2-3Galb1-3)GalNAca-Sp8
241	Neu5Aca2-6(Neu5Aca2-3Galb1-3)GalNAca-Sp14
242	Neu5Aca2-3Galb-Sp8
243	Neu5Aca2-3Galb1-3GalNAcb1-3Gala1-4Galb1-4Glcb-Sp0
244	Neu5Aca2-3Galb1-3GlcNAcb1-3Galb1-4GlcNAcb-Sp0
245	Fuca1-2(6S)Galb1-4Glcb-Sp0
246	Neu5Aca2-3Galb1-3GlcNAcb-Sp0
247	Neu5Aca2-3Galb1-4(6S)GlcNAcb-Sp8
248	Neu5Aca2-3Galb1-4(Fuca1-3)(6S)GlcNAcb-Sp8
249	Neu5Aca2-3Galb1-4(Fuca1-3)GlcNAcb1-3Galb1-4(Fuca1-3)GlcNAcb1-3Galb1-4(Fuca1-3)GlcNAcb-Sp0
250	Neu5Aca2-3Galb1-4(Fuca1-3)GlcNAcb-Sp0
251	Neu5Aca2-3Galb1-4(Fuca1-3)GlcNAcb-Sp8
252	Neu5Aca2-3Galb1-4(Fuca1-3)GlcNAcb1-3Galb-Sp8
253	Neu5Aca2-3Galb1-4(Fuca1-3)GlcNAcb1-3Galb1-4GlcNAcb-Sp8
254	Neu5Aca2-3Galb1-4GlcNAcb1-3Galb1-4GlcNAcb1-3Galb1-4GlcNAcb-Sp0
255	Neu5Aca2-3Galb1-4GlcNAcb-Sp0
256	Neu5Aca2-3Galb1-4GlcNAcb-Sp8
257	Neu5Aca2-3Galb1-4GlcNAcb1-3Galb1-4GlcNAcb-Sp0
258	Fuca1-2Galb1-4(6S)Glcb-Sp0
259	Neu5Aca2-3Galb1-4Glcb-Sp0
260	Neu5Aca2-3Galb1-4Glcb-Sp8
261	Neu5Aca2-6GalNAca-Sp8
262	Neu5Aca2-6GalNAcb1-4GlcNAcb-Sp0
263	Neu5Aca2-6Galb1-4(6S)GlcNAcb-Sp8
264	Neu5Aca2-6Galb1-4GlcNAcb-Sp0
265	Neu5Aca2-6Galb1-4GlcNAcb-Sp8
266	Neu5Aca2-6Galb1-4GlcNAcb1-3Galb1-4(Fuca1-3)GlcNAcb1-3Galb1-4(Fuca1-3)GlcNAcb-Sp0
267	Neu5Aca2-6Galb1-4GlcNAcb1-3Galb1-4GlcNAcb-Sp0
268	Neu5Aca2-6Galb1-4Glcb-Sp0
269	Neu5Aca2-6Galb1-4Glcb-Sp8
270	Neu5Aca2-6Galb-Sp8
271	Neu5Aca2-8Neu5Aca-Sp8
272	Neu5Aca2-8Neu5Aca2-3Galb1-4Glcb-Sp0
273	Galb1-3(Fuca1-4)GlcNAcb1-3Galb1-3(Fuca1-4)GlcNAcb-Sp0
274	Neu5Acb2-6GalNAca-Sp8
275	Neu5Acb2-6Galb1-4GlcNAcb-Sp8
276	Neu5Gca2-3Galb1-3(Fuca1-4)GlcNAcb-Sp0
277	Neu5Gca2-3Galb1-3GlcNAcb-Sp0
278	Neu5Gca2-3Galb1-4(Fuca1-3)GlcNAcb-Sp0
279	Neu5Gca2-3Galb1-4GlcNAcb-Sp0
280	Neu5Gca2-3Galb1-4Glcb-Sp0

281	Neu5Gca2-6GalNAca-Sp0
282	Neu5Gca2-6Galb1-4GlcNAcb-Sp0
283	Neu5Gca-Sp8
284	Neu5Aca2-3Galb1-4GlcNAcb1-6(Galb1-3)GalNAca-Sp14
285	Galb1-3GlcNAcb1-3Galb1-3GlcNAcb-Sp0
286	Galb1-4(Fuca1-3)(6S)GlcNAcb-Sp0
287	Galb1-4(Fuca1-3)(6S)Glcb-Sp0
288	Galb1-4(Fuca1-3)GlcNAcb1-3Galb1-3(Fuca1-4)GlcNAcb-Sp0
289	Galb1-4GlcNAcb1-3Galb1-3GlcNAcb-Sp0
290	Neu5Aca2-3Galb1-3GlcNAcb1-3Galb1-3GlcNAcb-Sp0
291	Neu5Aca2-3Galb1-4GlcNAcb1-3Galb1-3GlcNAcb-Sp0
292	4S(3S)Galb1-4GlcNAcb-Sp0
293	(6S)Galb1-4(6S)GlcNAcb-Sp0
294	(6P)Glcb-Sp10
295	Neu5Aca2-3Galb1-4(Fuca1-3)GlcNAcb1-6(Galb1-3)GalNAca-Sp14
296	Galb1-3Galb1-4GlcNAcb-Sp8
297	Neu5Aca2-6Galb1-4GlcNAcb1-2Mana1-6(Galb1-4GlcNAcb1-2Mana1-3)Manb1-4GlcNAcb1-4GlcNAcb-Sp12
298	Galb1-4GlcNAcb1-6(Galb1-4GlcNAcb1-3)Galb1-4GlcNAc-Sp0
299	GlcNAcb1-6(Galb1-4GlcNAcb1-3)Galb1-4GlcNAc-Sp0
300	Galb1-4GlcNAca1-6Galb1-4GlcNAcb-Sp0
301	Galb1-4GlcNAcb1-6Galb1-4GlcNAcb-Sp0
302	GalNAcb1-3Galb-Sp8
303	GlcAb1-3GlcNAcb-Sp8
304	Neu5Aca2-6Galb1-4GlcNAcb1-2Mana1-6(GlcNAcb1-2Mana1-3)Manb1-4GlcNAcb1-4GlcNAcb-Sp12
305	GlcNAcb1-3Man-Sp10
306	GlcNAcb1-4GlcNAcb-Sp10
307	GlcNAcb1-4GlcNAcb-Sp12
308	MurNAcb1-4GlcNAcb-Sp10
309	Mana1-6Manb-Sp10
310	Mana1-6(Mana1-3)Mana1-6(Mana1-3)Manb-Sp10
311	Mana1-2Mana1-6(Mana1-3)Mana1-6(Mana1-2Mana1-2Mana1-3)Mana-Sp9
312	Mana1-2Mana1-6(Mana1-2Mana1-3)Mana1-6(Mana1-2Mana1-2Mana1-3)Mana-Sp9
313	Neu5Aca2-3Galb1-4GlcNAcb1-6(Neu5Aca2-3Galb1-3)GalNAca-Sp14
314	Neu5Aca2-6Galb1-4GlcNAcb1-2Mana1-6(Neu5Aca2-3Galb1-4GlcNAcb1-2Mana1-3)Manb1-4GlcNAcb1-4GlcNAcb-Sp12
315	Galb1-4GlcNAcb1-2Mana1-6(Neu5Aca2-6Galb1-4GlcNAcb1-2Mana1-3)Manb1-4GlcNAcb1-4GlcNAcb-Sp12
316	Neu5Aca2-8Neu5Acb-Sp17
317	Neu5Aca2-8Neu5Aca2-8Neu5Acb-Sp8
318	Neu5Gcb2-6Galb1-4GlcNAc-Sp8
319	Galb1-3GlcNAcb1-2Mana1-6(Galb1-3GlcNAcb1-2Mana1-3)Manb1-4GlcNAcb1-4GlcNAcb-Sp19
320	Neu5Aca2-3Galb1-4GlcNAcb1-2Mana1-6(Neu5Aca2-3Galb1-4GlcNAcb1-2Mana1-3)Manb1-4GlcNAcb1-4GlcNAcb-Sp12
321	Neu5Aca2-3Galb1-4GlcNAcb1-2Mana1-6(Neu5Aca2-6Galb1-4GlcNAcb1-2Mana1-3)Manb1-4GlcNAcb1-4GlcNAcb-Sp12
322	Galb1-4(Fuca1-3)GlcNAcb1-2Mana1-6(Galb1-4(Fuca1-3)GlcNAcb1-2Mana1-3)Manb1-4GlcNAcb1-4GlcNAcb-Sp20
323	Neu5,9Ac2a2-3Galb1-3GlcNAcb-Sp0
324	Neu5Aca2-6Galb1-4GlcNAcb1-3Galb1-3GlcNAcb-Sp0
325	Neu5Aca2-3Galb1-3(Fuca1-4)GlcNAcb1-3Galb1-3(Fuca1-4)GlcNAcb-Sp0
326	Neu5Aca2-6Galb1-4GlcNAcb1-3Galb1-4GlcNAcb1-3Galb1-4GlcNAcb-Sp0
327	Gala1-4Galb1-4GlcNAcb1-3Galb1-4Glcb-Sp0
328	GalNAcb1-3Gala1-4Galb1-4GlcNAcb1-3Galb1-4Glcb-Sp0
329	GalNAca1-3(Fuca1-2)Galb1-4GlcNAcb1-3Galb1-4GlcNAcb-Sp0
330	GalNAca1-3(Fuca1-2)Galb1-4GlcNAcb1-3Galb1-4GlcNAcb1-3Galb1-4GlcNAcb-Sp0
331	Neu5Aca2-3Galb1-4(Fuca1-3)GlcNAcb1-6(Neu5Aca2-3Galb1-3)GalNAc-Sp14
332	GlcNAca1-4Galb1-4GlcNAcb1-3Galb1-4GlcNAcb1-3Galb1-4GlcNAcb-Sp0
333	GlcNAca1-4Galb1-4GlcNAcb-Sp0
334	GlcNAca1-4Galb1-3GlcNAcb-Sp0
335	GlcNAca1-4Galb1-4GlcNAcb1-3Galb1-4Glcb-Sp0
336	GlcNAca1-4Galb1-4GlcNAcb1-3Galb1-4(Fuca1-3)GlcNAcb1-3Galb1-4(Fuca1-3)GlcNAcb-Sp0
337	GlcNAca1-4Galb1-4GlcNAcb1-3Galb1-4GlcNAcb-Sp0
338	GlcNAca1-4Galb1-3GalNAc-Sp14
339	Neu5Aca2-6Galb1-4GlcNAcb1-2Mana1-6(Mana1-3)Manb1-4GlcNAcb1-4GlcNAc-Sp12
340	Mana1-6(Neu5Aca2-6Galb1-4GlcNAcb1-2Mana1-3)Manb1-4GlcNAcb1-4GlcNAc-Sp12
341	Neu5Aca2-6Galb1-4GlcNAcb1-2Mana1-6Manb1-4GlcNAcb1-4GlcNAc-Sp12
342	Neu5Aca2-6Galb1-4GlcNAcb1-2Mana1-3Manb1-4GlcNAcb1-4GlcNAc-Sp12
343	Galb1-4GlcNAcb1-2Mana1-3Manb1-4GlcNAcb1-4GlcNAc-Sp12
344	Galb1-4GlcNAcb1-2Mana1-6Manb1-4GlcNAcb1-4GlcNAc-Sp12
345	Mana1-6(Galb1-4GlcNAcb1-2Mana1-3)Manb1-4GlcNAcb1-4GlcNAcb-Sp12
346	GlcNAcb1-2Mana1-6(GlcNAcb1-2Mana1-3)Manb1-4GlcNAcb1-4(Fuca1-6)GlcNAcb-Sp22
347	Galb1-4GlcNAcb1-2Mana1-6(Galb1-4GlcNAcb1-2Mana1-3)Manb1-4GlcNAcb1-4(Fuca1-6)GlcNAcb-Sp22
348	Galb1-3GlcNAcb1-2Mana1-6(Galb1-3GlcNAcb1-2Mana1-3)Manb1-4GlcNAcb1-4(Fuca1-6)GlcNAcb-Sp22

349	(6S)GlcNAcb1-3Galb1-4GlcNAcb-Sp0
350	KDNa2-3Galb1-4(Fuca1-3)GlcNAc-Sp0
351	KDNa2-6Galb1-4GlcNAc-Sp0
352	KDNa2-3Galb1-4Glc-Sp0
353	KDNa2-3Galb1-3GalNAca-Sp14
354	Fuca1-2Galb1-3GlcNAcb1-2Mana1-6(Fuca1-2Galb1-3GlcNAcb1-2Mana1-3)Manb1-4GlcNAcb1-4GlcNAcb-Sp20
355	Fuca1-2Galb1-4GlcNAcb1-2Mana1-6(Fuca1-2Galb1-4GlcNAcb1-2Mana1-3)Manb1-4GlcNAcb1-4GlcNAcb-Sp20
356	Fuca1-2Galb1-4(Fuca1-3)GlcNAcb1-2Mana1-6(Fuca1-2Galb1-4(Fuca1-3)GlcNAcb1-2Mana1-3)Manb1-4GlcNAcb1-4GlcNAb-Sp20
357	Gala1-3Galb1-4GlcNAcb1-2Mana1-6(Gala1-3Galb1-4GlcNAcb1-2Mana1-3)Manb1-4GlcNAcb1-4GlcNAcb-Sp20
358	Galb1-4GlcNAcb1-2Mana1-6(Mana1-3)Manb1-4GlcNAcb1-4GlcNAcb-Sp12
359	Fuca1-4(Galb1-3)GlcNAcb1-2Mana1-6(Fuca1-4(Galb1-3)GlcNAcb1-2Mana1-3)Manb1-4GlcNAcb1-4(Fuca1-6)GlcNAcb-Sp22
360	Neu5Aca2-6GlcNAcb1-4GlcNAc-Sp21
361	Neu5Aca2-6GlcNAcb1-4GlcNAcb1-4GlcNAc-Sp21
362	Galb1-4(Fuca1-3)GlcNAcb1-6(Fuca1-2Galb1-4GlcNAcb1-3)Galb1-4Glc-Sp21
363	Galb1-4GlcNAcb1-2Mana1-6(Galb1-4GlcNAcb1-4(Galb1-4GlcNAcb1-2)Mana1-3)Manb1-4GlcNAcb1-4GlcNAc-Sp21
364	GalNAca1-3(Fuca1-2)Galb1-4GlcNAcb1-2Mana1-6(GalNAca1-3(Fuca1-2)Galb1-4GlcNAcb1-2Mana1-3)Manb1-4GlcNAcb1-4GlcNAcb-Sp20
365	Gala1-3(Fuca1-2)Galb1-4GlcNAcb1-2Mana1-6(Gala1-3(Fuca1-2)Galb1-4GlcNAcb1-2Mana1-3)Manb1-4GlcNAcb1-4GlcNAcb-Sp20
366	Gala1-3Galb1-4(Fuca1-3)GlcNAcb1-2Mana1-6(Gala1-3Galb1-4(Fuca1-3)GlcNAcb1-2Mana1-3)Manb1-4GlcNAcb1-4GlcNAcb-Sp20
367	GalNAca1-3(Fuca1-2)Galb1-3GlcNAcb1-2Mana1-6(GalNAca1-3(Fuca1-2)Galb1-3GlcNAcb1-2Mana1-3)Manb1-4GlcNAcb1-4GlcNAcb-Sp20
368	Galα1-3(Fucα1-2)Galβ1-3GlcNAcβ1-2Manα1-6(Galα1-3(Fucα1-2)Galβ1-3GlcNAcβ1-2Manα1-3)Manβ1-4GlcNAcβ1-4GlcNAcβ-Sp20
369	Fuca1-4(Fuca1-2Galb1-3)GlcNAcb1-2Mana1-3(Fuca1-4(Fuca1-2Galb1-3)GlcNAcb1-2Mana1-3)Manb1-4GlcNAcb1-4GlcNAcb-Sp19
370	Neu5Aca2-3Galb1-4GlcNAcb1-3GalNAc-Sp14
371	Neu5Aca2-6Galb1-4GlcNAcb1-3GalNAc-Sp14
372	Neu5Aca2-3Galb1-4(Fuca1-3)GlcNAcb1-3GalNAca-Sp14
373	GalNAcb1-4GlcNAcb1-2Mana1-6(GalNAcb1-4GlcNAcb1-2Mana1-3)Manb1-4GlcNAcb1-4GlcNAc-Sp12
374	Galb1-3GalNAca1-3(Fuca1-2)Galb1-4Glc-Sp0
375	Galb1-3GalNAca1-3(Fuca1-2)Galb1-4GlcNAc-Sp0
376	Galb1-3GlcNAcb1-3Galb1-4GlcNAcb1-6(Galb1-3GlcNAcb1-3)Galb1-4Glcb-Sp0
377	Galb1-4(Fuca1-3)GlcNAcb1-6(Galb1-3GlcNAcb1-3)Galb1-4Glc-Sp21
378	Galb1-4GlcNAcb1-6(Fuca1-4(Fuca1-2Galb1-3)GlcNAcb1-3)Galb1-4Glc-Sp21
379	Galb1-4(Fuca1-3)GlcNAcb1-6(Fuca1-4(Fuca1-2Galb1-3)GlcNAcb1-3)Galb1-4Glc-Sp21
380	Galb1-3GlcNAcb1-3Galb1-4(Fuca1-3)GlcNAcb1-6(Galb1-3GlcNAcb1-3)Galb1-4Glc-Sp21
381	Galb1-4GlcNAcb1-6(Galb1-4GlcNAcb1-2)Mana1-6(Galb1-4GlcNAcb1-4(Galb1-4GlcNAcb1-2)Mana1-3)Manb1-4GlcNAcb1-4GlcNAcb-Sp21
382	GlcNAcb1-2Mana1-6(GlcNAcb1-4(GlcNAcb1-2)Mana1-3)Manb1-4GlcNAcb1-4GlcNAc-Sp21
383	Fuca1-2Galb1-3GalNAca1-3(Fuca1-2)Galb1-4Glcb-Sp0
384	Fuca1-2Galb1-3GalNAca1-3(Fuca1-2)Galb1-4GlcNAcb-Sp0
385	Galb1-3GlcNAcb1-3GalNAca-Sp14
386	GalNAcb1-4(Neu5Aca2-3)Galb1-4GlcNAcb1-3GalNAca-Sp14
387	GalNAca1-3(Fuca1-2)Galb1-3GalNAca1-3(Fuca1-2)Galb1-4GlcNAcb-Sp0
388	Gala1-3Galb1-3GlcNAcb1-2Mana1-6(Gala1-3Galb1-3GlcNAcb1-2Mana1-3)Manb1-4GlcNAcb1-4GlcNAc-Sp19
389	Gala1-3Galb1-3(Fuca1-4)GlcNAcb1-2Mana1-6(Gala1-3Galb1-3(Fuca1-4)GlcNAcb1-2Mana1-3)Manb1-4GlcNAcb1-4GlcNAc-Sp19
390	GlcNAcb1-2Mana1-6(Galb1-4GlcNAcb1-2Mana1-3)Manb1-4GlcNAcb1-4GlcNAc-Sp12
391	Galb1-4GlcNAcb1-2Mana1-6(GlcNAcb1-2Mana1-3)Manb1-4GlcNAcb1-4GlcNAc-Sp12
392	Neu5Aca2-3Galb1-3GlcNAcb1-3GalNAca-Sp14
393	Fuca1-2Galb1-4GlcNAcb1-3GalNAca-Sp14
394	Galb1-4(Fuca1-3)GlcNAcb1-3GalNAca-Sp14
395	GalNAca1-3GalNAcb1-3Gala1-4Galb1-4GlcNAcb-Sp0
396	Gala1-4Galb1-3GlcNAcb1-2Mana1-6(Gala1-4Galb1-3GlcNAcb1-2Mana1-3)Manb1-4GlcNAcb1-4GlcNAcb-Sp19
397	Gala1-4Galb1-4GlcNAcb1-2Mana1-6(Gala1-4Galb1-4GlcNAcb1-2Mana1-3)Manb1-4GlcNAcb1-4GlcNAcb-Sp24
398	Gala1-3Galb1-4GlcNAcb1-3GalNAca-Sp14
399	Galb1-3GlcNAcb1-6Galb1-4GlcNAcb-Sp0
400	Galb1-3GlcNAca1-6Galb1-4GlcNAcb-Sp0
401	GalNAcb1-3Gala1-6Galb1-4Glcb-Sp8
402	Gala1-3(Fuca1-2)Galb1-4(Fuca1-3)Glcb-Sp21
403	Galb1-4GlcNAcb1-6(Neu5Aca2-6Galb1-3GlcNAcb1-3)Galb1-4Glc-Sp21

404	Galb1-3GalNAcb1-4(Neu5Aca2-8Neu5Aca2-3)Galb1-4Glcb-Sp0
405	Neu5Aca2-3Galb1-3GalNAcb1-4(Neu5Aca2-8Neu5Aca2-3)Galb1-4Glcb-Sp0
406	Gala1-3(Fuca1-2)Galb1-4GlcNAcb1-3GalNAca-Sp14
407	GalNAca1-3(Fuca1-2)Galb1-4GlcNAcb1-3GalNAca-Sp14
408	GalNAca1-3GalNAcb1-3Gala1-4Galb1-4Glcb-Sp0
409	Fuca1-2Galb1-4(Fuca1-3)GlcNAcb1-3GalNAca-Sp14
410	Gala1-3(Fuca1-2)Galb1-4(Fuca1-3)GlcNAcb1-3GalNAc-Sp14
411	GalNAca1-3(Fuca1-2)Galb1-4(Fuca1-3)GlcNAcb1-3GalNAc-Sp14
412	Galb1-4(Fuca1-3)GlcNAcb1-2Mana1-6(Galb1-4(Fuca1-3)GlcNAcb1-2Mana1-3)Manb1-4GlcNAcb1-4(Fuca1-6)GlcNAcb-Sp22
413	Fuca1-2Galb1-4GlcNAcb1-2Mana1-6(Fuca1-2Galb1-4GlcNAcb1-2Mana1-3)Manb1-4GlcNAcb1-4(Fuca1-6)GlcNAcb-Sp22
414	GlcNAcb1-2(GlcNAcb1-6)Mana1-6(GlcNAcb1-2Mana1-3)Manb1-4GlcNAcb1-4GlcNAcb-Sp19
415	Fuca1-2Galb1-3GlcNAcb1-3GalNAc-Sp14
416	Gala1-3(Fuca1-2)Galb1-3GlcNAcb1-3GalNAc-Sp14
417	GalNAca1-3(Fuca1-2)Galb1-3GlcNAcb1-3GalNAc-Sp14
418	Gala1-3Galb1-3GlcNAcb1-3GalNAc-Sp14
419	Fuca1-2Galb1-3GlcNAcb1-2Mana1-6(Fuca1-2Galb1-3GlcNAcb1-2Mana1-3)Manb1-4GlcNAcb1-4(Fuca1-6)GlcNAcb-Sp22
420	Gala1-3(Fuca1-2)Galb1-4GlcNAcb1-2Mana1-6(Gala1-3(Fuca1-2)Galb1-4GlcNAcb1-2Mana1-3)Manb1-4GlcNAcb1-4(Fuca1-6)GlcNAcb-Sp22
421	Galb1-3GlcNAcb1-6(Galb1-3GlcNAcb1-2)Mana1-6(Galb1-3GlcNAcb1-2Mana1-3)Manb1-4GlcNAcb1-4GlcNAcb-Sp19
422	Galb1-4GlcNAcb1-6(Fuca1-2Galb1-3GlcNAcb1-3)Galb1-4Glc-Sp21
423	Fuca1-3GlcNAcb1-6(Galb1-4GlcNAcb1-3)Galb1-4Glc-Sp21
424	GlcNAcb1-2Mana1-6(GlcNAcb1-4)(GlcNAcb1-2Mana1-3)Manb1-4GlcNAcb1-4GlcNAc-Sp21
425	GlcNAcb1-2Mana1-6(GlcNAcb1-4)(GlcNAcb1-4(GlcNAcb1-2)Mana1-3)Manb1-4GlcNAcb1-4GlcNAc-Sp21
426	GlcNAcb1-6(GlcNAcb1-2)Mana1-6(GlcNAcb1-4)(GlcNAcb1-2Mana1-3)Manb1-4GlcNAcb1-4GlcNAc-Sp21
427	GlcNAcb1-6(GlcNAcb1-2)Mana1-6(GlcNAcb1-4)(GlcNAcb1-4(GlcNAcb1-2)Mana1-3)Manb1-4GlcNAcb1-4GlcNAc-Sp21
428	Galb1-4GlcNAcb1-2Mana1-6(GlcNAcb1-4)(Galb1-4GlcNAcb1-2Mana1-3)Manb1-4GlcNAcb1-4GlcNAc-Sp21
429	Galb1-4GlcNAcb1-2Mana1-6(GlcNAcb1-4)(Galb1-4GlcNAcb1-4(Galb1-4GlcNAcb1-2)Mana1-3)Manb1-4GlcNAcb1-4GlcNAc-Sp21
430	Galb1-4GlcNAcb1-6(Galb1-4GlcNAcb1-2)Mana1-6(GlcNAcb1-4)(Galb1-4GlcNAcb1-2Mana1-3)Manb1-4GlcNAcb1-4GlcNAc-Sp21
431	Galb1-4GlcNAcb1-6(Galb1-4GlcNAcb1-2)Mana1-6(GlcNAcb1-4)(Galb1-4GlcNAcb1-4(Galb1-4GlcNAcb1-2)Mana1-3)Manb1-4GlcNAcb1-4GlcNAc-Sp21
432	Galb1-4Galb-Sp10
433	Galb1-6Galb-Sp10
434	Neu5Aca2-3Galb1-4GlcNAcb1-3Galb-Sp8
435	GalNAcb1-6GalNAcb-Sp8
436	(6S)Galb1-3GlcNAcb-Sp0
437	(6S)Galb1-3(6S)GlcNAc-Sp0
438	Fuca1-2Galb1-4 GlcNAcb1-2Mana1-6(Fuca1-2Galb1-4GlcNAcb1-2(Fuca1-2Galb1-4GlcNAcb1-4)Mana1-3)Manb1-4GlcNAcb1-4GlcNAcb-Sp12
439	Fuca1-2Galb1-4(Fuca1-3)GlcNAcb1-2Mana1-6(Fuca1-2Galb1-4(Fuca1-3)GlcNAcb1-4(Fuca1-2Galb1-4(Fuca1-3)GlcNAcb1-2)Mana1-3)Manb1-4GlcNAcb1-4GlcNAcb-Sp12
440	Galb1-4(Fuca1-3)GlcNAcb1-6GalNAc-Sp14
441	Galb1-4GlcNAcb1-2Mana-Sp0
442	Fuca1-2Galb1-4GlcNAcb1-6(Fuca1-2Galb1-4GlcNAcb1-3)GalNAc-Sp14
443	Gala1-3(Fuca1-2)Galb1-4GlcNAcb1-6(Gala1-3(Fuca1-2)Galb1-4GlcNAcb1-3)GalNAc-Sp14
444	GalNAca1-3(Fuca1-2)Galb1-4GlcNAcb1-6(GalNAca1-3(Fuca1-2)Galb1-4GlcNAcb1-3)GalNAc-Sp14
445	Neu5Aca2-8Neu5Aca2-3Galb1-3GalNAcb1-4(Neu5Aca2-8Neu5Aca2-3)Galb1-4Glcb-Sp0
446	GalNAcb1-4Galb1-4Glcb-Sp0
447	GalNAca1-3(Fuca1-2)Galb1-4GlcNAcb1-2Mana1-6(GalNAca1-3(Fuca1-2)Galb1-4GlcNAcb1-2Mana1-3)Manb1-4GlcNAcb1-4(Fuca1-6)GlcNAcb-Sp22
448	Gala1-3(Fuca1-2)Galb1-3GlcNAcb1-2Mana1-6(Gala1-3(Fuca1-2)Galb1-3GlcNAcb1-2Mana1-3)Manb1-4GlcNAcb1-4(Fuca1-6)GlcNAcb-Sp22
449	Neu5Aca2-6Galb1-4GlcNAcb1-6(Fuca1-2Galb1-3GlcNAcb1-3)Galb1-4Glc-Sp21
450	GalNAca1-3(Fuca1-2)Galb1-3GlcNAcb1-2Mana1-6(GalNAca1-3(Fuca1-2)Galb1-3GlcNAcb1-2Mana1-3)Manb1-4GlcNAcb1-4(Fuca1-6)GlcNAcb-Sp22
451	Galb1-4GlcNAcb1-6(Galb1-4GlcNAcb1-2)Mana1-6(Galb1-4GlcNAcb1-2Mana1-3)Manb1-4GlcNAcb1-4GlcNAcb-Sp19
452	Neu5Aca2-3Galb1-4GlcNAcb1-2Mana1-6(GlcNAcb1-4)(Neu5Aca2-3Galb1-4GlcNAcb1-2Mana1-3)Manb1-4GlcNAcb1-4GlcNAcb-Sp21
453	Neu5Aca2-3Galb1-4GlcNAcb1-4Mana1-6(GlcNAcb1-4)(Neu5Aca2-3Galb1-4GlcNAcb1-4(Neu5Aca2-3Galb1-4GlcNAcb1-2)Mana1-3)Manb1-4GlcNAcb1-4GlcNAcb-Sp21
454	Neu5Aca2-3Galb1-4GlcNAcb1-6(Neu5Aca2-3Galb1-4GlcNAcb1-2)Mana1-6(GlcNAcb1-4)(Neu5Aca2-3Galb1-4GlcNAcb1-2Mana1-3)Manb1-4GlcNAcb1-4GlcNAcb-Sp21
455	Neu5Aca2-3Galb1-4GlcNAcb1-6(Neu5Aca2-3Galb1-4GlcNAcb1-2)Mana1-6(GlcNAcb1-4)(Neu5Aca2-3Galb1-4GlcNAcb1-4(Neu5Aca2-3Galb1-4GlcNAcb1-2)Mana1-3)Manb1-4GlcNAcb1-4GlcNAcb-Sp21

456	Neu5Aca2-6Galb1-4GlcNAcb1-2Mana1-6(GlcNAcb1-4)(Neu5Aca2-6Galb1-4GlcNAcb1-2Mana1-3)Manb1-4GlcNAcb1-4GlcNAcb-Sp21
457	Neu5Aca2-6Galb1-4GlcNAcb1-4Mana1-6(GlcNAcb1-4)(Neu5Aca2-6Galb1-4GlcNAcb1-4(Neu5Aca2-6Galb1-4GlcNAcb1-2)Mana1-3)Manb1-4GlcNAcb1-4GlcNAcb-Sp21
458	Neu5Aca2-6Galb1-4GlcNAcb1-6(Neu5Aca2-6Galb1-4GlcNAcb1-2)Mana1-6(GlcNAcb1-4)(Neu5Aca2-6Galb1-4GlcNAcb1-2Mana1-3)Manb1-4GlcNAcb1-4GlcNAcb-Sp21
459	Neu5Aca2-6Galb1-4GlcNAcb1-6(Neu5Aca2-6Galb1-4GlcNAcb1-2)Mana1-6(GlcNAcb1-4)(Neu5Aca2-6Galb1-4GlcNAcb1-4(Neu5Aca2-6Galb1-4GlcNAcb1-2)Mana1-3)Manb1-4GlcNAcb1-4GlcNAcb-Sp21
460	Gala1-3(Fuca1-2)Galb1-3GalNAca-Sp8
461	Gala1-3(Fuca1-2)Galb1-3GalNAcb-Sp8
462	Glca1-6Glca1-6Glca1-6Glcb-Sp10
463	Glca1-4Glca1-4Glca1-4Glcb-Sp10
464	Neu5Aca2-3Galb1-4GlcNAcb1-6(Neu5Aca2-3Galb1-4GlcNAcb1-3)GalNAca-Sp14
465	Fuca1-2Galb1-4(Fuca1-3)GlcNAcb1-2Mana1-6(Fuca1-2Galb1-4(Fuca1-3)GlcNAcb1-2Mana1-3)Manb1-4GlcNAcb1-4(Fuca1-6)GlcNAcb-Sp24
466	Fuca1-2Galb1-3(Fuca1-4)GlcNAcb1-2Mana1-6(Fuca1-2Galb1-3(Fuca1-4)GlcNAcb1-2Mana1-3)Manb1-4GlcNAcb1-4(Fuca1-6)GlcNAcb1-4(Fuca1-6)GlcNAcb-Sp19
467	GlcNAcb1-6(GlcNAcb1-2)Mana1-6(GlcNAcb1-2Mana1-3)Manb1-4GlcNAcb1-4(Fuca1-6)GlcNAcb-Sp24
468	Galb1-3GlcNAcb1-2Mana1-6(GlcNAcb1-4)(Galb1-3GlcNAcb1-2Mana1-3)Manb1-4GlcNAcb1-4GlcNAcb-Sp21
469	Neu5Aca2-6Galb1-4GlcNAcb1-6(Galb1-3GlcNAcb1-3)Galb1-4Glcb-Sp21
470	Neu5Aca2-3Galb1-4GlcNAcb1-2Mana-Sp0
471	Neu5Aca2-3Galb1-4GlcNAcb1-6GalNAca-Sp14
472	Neu5Aca2-6Galb1-4GlcNAcb1-6GalNAca-Sp14
473	Neu5Aca2-6Galb1-4 GlcNAcb1-6(Neu5Aca2-6Galb1-4GlcNAcb1-3)GalNAca-Sp14
474	Neu5Aca2-6Galb1-4GlcNAcb1-2Mana1-6(Neu5Aca2-6Galb1-4GlcNAcb1-2Mana1-3)Manb1-4GlcNAcb1-4(Fuca1-6)GlcNAcb-Sp24
475	Neu5Aca2-3Galb1-4GlcNAcb1-2Mana1-6(Neu5Aca2-3Galb1-4GlcNAcb1-2Mana1-3)Manb1-4GlcNAcb1-4(Fuca1-6)GlcNAcb-Sp24
476	Mana1-6(Mana1-3)Manb1-4GlcNAcb1-4(Fuca1-6)GlcNAcb-Sp19
477	Galb1-4GlcNAcb1-6(Galb1-4GlcNAcb1-2)Mana1-6(Galb1-4GlcNAcb1-2Mana1-3)Manb1-4GlcNAcb1-4(Fuca1-6)GlcNAcb-Sp24
478	Neu5Aca2-3Galb1-3GlcNAcb1-2Mana1-6(GlcNAcb1-4)(Neu5Aca2-3Galb1-3GlcNAcb1-2Mana1-3)Manb1-4GlcNAcb1-4GlcNAc-Sp21
479	Neu5Aca2-6Galb1-4GlcNAcb1-6(Fuca1-2Galb1-4(Fuca1-3)GlcNAcb1-3)Galb1-4Glc-Sp21
480	Galb1-3GlcNAcb1-6GalNAca-Sp14
481	Gala1-3Galb1-3GlcNAcb1-6GalNAca-Sp14
482	Galb1-3(Fuca1-4)GlcNAcb1-6GalNAca-Sp14
483	Neu5Aca2-3Galb1-3GlcNAcb1-6GalNAca-Sp14
484	(3S)Galb1-3(Fuca1-4)GlcNAcb-Sp0
485	Galb1-4(Fuca1-3)GlcNAcb1-6(Neu5Aca2-6(Neu5Aca2-3Galb1-3)GlcNAcb1-3)Galb1-4Glc-Sp21
486	Fuca1-2Galb1-4GlcNAcb1-6GalNAca-Sp14
487	Gala1-3Galb1-4GlcNAcb1-6GalNAca-Sp14
488	Galb1-4(Fuca1-3)GlcNAcb1-2Mana-Sp0
489	Fuca1-2(6S)Galb1-3GlcNAcb-Sp0
490	Gala1-3(Fuca1-2)Galb1-4GlcNAcb1-6GalNAca-Sp14
491	Fuca1-2Galb1-4GlcNAcb1-2Mana-Sp0
492	Fuca1-2Galb1-3(6S)GlcNAcb-Sp0
493	Fuca1-2(6S)Galb1-3(6S)GlcNAcb-Sp0
494	Neu5Aca2-6GalNAcb1-4(6S)GlcNAcb-Sp8
495	GalNAcb1-4(Fuca1-3)(6S)GlcNAcb-Sp8
496	(3S)GalNAcb1-4(Fuca1-3)GlcNAcb-Sp8
497	Fuca1-2Galb1-3GlcNAcb1-6(Fuca1-2Galb1-3GlcNAcb1-3)GalNAca-Sp14
498	GalNAca1-3(Fuca1-2)Galb1-3GlcNAcb1-6GalNAca-Sp14
499	GlcNAcb1-6(GlcNAcb1-2)Mana1-6(GlcNAcb1-4)(GlcNAcb1-4(GlcNAcb1-2)Mana1-3)Manb1-4GlcNAcb1-4(Fuca1-6)GlcNAc-Sp21
500	Galb1-4GlcNAcb1-6(Galb1-4GlcNAcb1-2)Mana1-6(GlcNAcb1-4)Galb1-4GlcNAcb1-4(Gal b1-4GlcNAcb1-2)Mana1-3)Manb1-4GlcNAcb1-4(Fuca1-6)GlcNAc-Sp21
501	Galb1-3GlcNAca1-3Galb1-4GlcNAcb-Sp8
502	Galb1-3(6S)GlcNAcb-Sp8
503	(6S)(4S)GalNAcb1-4GlcNAc-Sp8
504	(6S)GalNAcb1-4GlcNAc-Sp8
505	(3S)GalNAcb1-4(3S)GlcNAc-Sp8
506	GalNAcb1-4(6S)GlcNAc-Sp8
507	(3S)GalNAcb1-4GlcNAc-Sp8
508	(4S)GalNAcb-Sp10
509	Galb1-4(6P)GlcNAcb-Sp0
510	(6P)Galb1-4GlcNAcb-SP0
511	GalNAca1-3(Fuca1-2)Galb1-4GlcNAcb1-6GalNAc-Sp14
512	Neu5Aca2-6Galb1-4GlcNAcb1-2Man-Sp0
513	Gala1-3Galb1-4GlcNAcb1-2Mana-Sp0
514	Gala1-3(Fuca1-2)Galb1-4GlcNAcb1-2Mana-Sp0

515	GalNAca1-3(Fuca1-2)Galb1-4 GlcNAcb1-2Mana-Sp0
516	Galb1-3GlcNAcb1-2Mana-Sp0
517	Gala1-3(Fuca1-2)Galb1-3GlcNAcb1-6GalNAc-Sp14
518	Neu5Aca2-3Galb1-3GlcNAcb1-2Mana-Sp0
519	Gala1-3Galb1-3GlcNAcb1-2Mana-Sp0
520	GalNAcb1-4GlcNAcb1-2Mana-Sp0
521	Neu5Aca2-3Galb1-3GalNAcb1-4Galb1-4Glcb-Sp0
522	GlcNAcb1-2 Mana1-6(GlcNAcb1-4)(GlcNAcb1-2Mana1-3)Manb1-4GlcNAcb1-4(Fuca1-6)GlcNAc-Sp21
523	Galb1-4GlcNAcb1-2 Mana1-6(GlcNAcb1-4)(Galb1-4GlcNAcb1-2Mana1-3)Manb1-4GlcNAcb1-4(Fuca1-6)GlcNAc-Sp21
524	Galb1-4GlcNAcb1-2 Mana1-6(Galb1-4GlcNAcb1-4)(Galb1-4GlcNAcb1-2Mana1-3)Manb1-4GlcNAcb1-4(Fuca1-6)GlcNAc-Sp21
525	Fuca1-4(Galb1-3)GlcNAcb1-2 Mana-Sp0
526	Neu5Aca2-3Galb1-4(Fuca1-3)GlcNAcb1-2Mana-Sp0
527	GlcNAcb1-3Galb1-4GlcNAcb1-6(GlcNAcb1-3)Galb1-4GlcNAc-Sp0
528	GalNAca1-3(Fuca1-2)Galb1-3GalNAcb1-3Gala1-4Galb1-4Glc-Sp21
529	Gala1-3(Fuca1-2)Galb1-3GalNAcb1-3Gala1-4Galb1-4Glc-Sp21
530	Galb1-3GalNAcb1-3Gal-Sp21
531	GlcNAcb1-3Galb1-4GlcNAcb1-2Mana1-6(GlcNAcb1-3Galb1-4GlcNAcb1-2Mana1-3)Manb1-4GlcNAcb1-4GlcNAcb-Sp12
532	GlcNAcb1-3Galb1-4GlcNAcb1-2Mana1-6(GlcNAcb1-3Galb1-4GlcNAcb1-2Mana1-3)Manb1-4GlcNAcb1-4GlcNAcb-Sp25
533	Galβ1-4GlcNAcβ1-3Galβ1-4GlcNAcβ1-2Manα1-6(Galβ1-4GlcNAcβ1-3Galβ1-4GlcNAcβ1-2Manα1-3)Manβ1-4GlcNAcβ1-4GlcNAcβ-Sp12
534	Fuca1-2Galb1-4GlcNAcb1-3Galb1-4GlcNAcb1-2Mana1-6(Fuca1-2Galb1-4GlcNAcb1-3Galb1-4GlcNAcb1-2Mana1-3)Manb1-4GlcNAcb1-4GlcNAcb-Sp24
535	GlcNAcb1-3Galb1-4GlcNAcb1-3Galb1-4GlcNAcb1-2Mana1-6(GlcNAcb1-3Galb1-4GlcNAcb1-3Galb1-4GlcNAcb1-2Mana1-3)Manb1-4GlcNAcb1-4GlcNAcb-Sp12
536	GlcNAcb1-3Galb1-4GlcNAcb1-3Galb1-4GlcNAcb1-2Mana1-6(GlcNAcb1-3Galb1-4GlcNAcb1-3Galb1-4GlcNAcb1-2Mana1-3)Manb1-4GlcNAcb1-4GlcNAcb-Sp25
537	Galb1-4GlcNAcb1-3Galb1-4GlcNAcb1-3Galb1-4GlcNAcb1-2Mana1-6(Galb1-4GlcNAcb1-3Galb1-4GlcNAcb1-3Galb1-4GlcNAcb1-2Mana1-3)Manb1-4GlcNAcb1-4GlcNAcb-Sp12
538	Galb1-3GlcNAcb1-3Galb1-4GlcNAcb1-2Mana1-6(Galb1-3GlcNAcb1-3Galb1-4GlcNAcb1-2Mana1-3)Manb1-4GlcNAcb1-4GlcNAc-Sp25
539	Neu5Gca2-8Neu5Gca2-3Galb1-4GlcNAc-Sp0
540	Neu5Aca2-8Neu5Gca2-3Galb1-4GlcNAc-Sp0
541	Neu5Gca2-8Neu5Aca2-3Galb1-4GlcNAc-Sp0
542	Neu5Gca2-8Neu5Gca2-3Galb1-4GlcNAcb1-3Galb1-4GlcNAc-Sp0
543	Neu5Gca2-8Neu5Gca2-6Galb1-4GlcNAc-Sp0
544	Neu5Aca2-8Neu5Aca2-3Galb1-4GlcNAc-Sp0
545	GlcNAcb1-3Galb1-4GlcNAcb1-6(GlcNAcb1-3Galb1-4GlcNAcb1-2)Mana1-6(GlcNAcb1-3Galb1-4GlcNAcb1-2Man a1-3)Manb1-4GlcNAcb1-4GlcNAc-Sp24
546	Galb1-4GlcNAcb1-3Galb1-4GlcNAcb1-6(Galb1-4GlcNAcb1-3Galb1-4GlcNAcb1-2)Mana1-6(Galb1-4GlcNAcb1-3Galb1-4GlcNAcb1-2Mana1-3)Mana1-4GlcNAcb1-4GlcNAc-Sp24
547	Gala1-3Galb1-4GlcNAcb1-2Mana1-6(Gala1-3Galb1-4GlcNAcb1-2Mana1-3)Manb1-4GlcNAcb1-4GlcNAc-Sp24
548	GlcNAcb1-3Galb1-4GlcNAcb1-6(GlcNAcb1-3Galb1-3)GalNAca-Sp14
549	GalNAcb1-3GlcNAcb-Sp0
550	GalNAcb1-4GlcNAcb1-3GalNAcb1-4GlcNAcb-Sp0
551	GlcNAcb1-3Galb1-4GlcNAcb1-3Galb1-4GlcNAcb1-3Galb1-4GlcNAcb1-3Galb1-4GlcNAcb1-2Mana1-6(GlcNAcb1-3Galb1-4GlcNAcb1-3Galb1-4GlcNAcb1-3Galb1-4GlcNAcb1-3Galb1-4GlcNAcb1-2Mana1-3)Manb1-4GlcNAcb1-4GlcNAcb-Sp25
552	Galb1-4GlcNAcb1-3Galb1-4GlcNAcb1-3Galb1-4GlcNAcb1-3Galb1-4GlcNAcb1-3Galb1-4GlcNAcb1-2Mana1-6(Galb1-4GlcNAcb1-3Galb1-4GlcNAcb1-3Galb1-4GlcNAcb1-3Galb1-4GlcNAcb1-3Galb1-4GlcNAcb1-2Mana1-3)Manb1-4GlcNAcb1-4GlcNAcb-Sp25
553	GlcNAb1-3Galb1-3GalNAc-Sp14
554	Galb1-3GlcNAcb1-6(Galb1-3)GalNAc-Sp14
555	(3S)GlcAb1-3Galb1-4GlcNAcb1-3Galb1-4Glc-Sp0
556	(3S)GlcAb1-3Galb1-4GlcNAcb1-2Mana-Sp0
557	Galb1-3GlcNAcb1-3Galb1-4GlcNAcb1-3Galb1-4GlcNAcb1-6(Galb1-3GlcNAcb1-3Galb1-4GlcNAcb1-3Galb1-4GlcNAb1-2)Mana1-6(Galb1-3GlcNAcb1-3Galb1-4GlcNAcb1-3Galb1-4GlcNAcb1-2Mana1-3)Manb1-4GlcNAcb1-4(Fuca1-6)GlcNAcb-Sp24
558	Galb1-3GlcNAcb1-3Galb1-4GlcNAcb1-6(Galb1-3GlcNAcb1-3Galb1-4GlcNAb1-2)Mana1-6(Galb1-3GlcNAcb1-3Galb1-4GlcNAcb1-2Mana1-3)Manb1-4GlcNAcb1-4(Fuca1-6)GlcNAcb-Sp24
559	Neu5Aca2-8Neu5Aca2-3Galb1-3GalNAcb1-4(Neu5Aca2-3)Galb1-4Glc-Sp21
560	Galb1-4GlcNAcb1-3Galb1-4GlcNAcb1-2Mana1-6(Galb1-4GlcNAcb1-3Galb1-4GlcNAcb1-2Mana1-3)Manb1-4GlcNAcb1-4(Fuca1-6)GlcNAcb-Sp24
561	GlcNAcb1-3Galb1-4GlcNAcb1-3Galb1-4GlcNAcb1-2Mana1-6(GlcNAcb1-3Galb1-4GlcNAcb1-3Galb1-4GlcNAcb1-2Mana1-3)Manb1-4GlcNAcb1-4(Fuca1-6)GlcNAcb-Sp24
562	Galb1-4GlcNAcb1-3Galb1-4GlcNAcb1-6(Galb1-4GlcNAcb1-3Galb1-4GlcNAb1-2)Mana1-6(Galb1-4GlcNAcb1-3Galb1-4GlcNAcb1-2Mana1-3)Manb1-4GlcNAcb1-4(Fuca1-6)GlcNAcb-Sp24

563	Galb1-4GlcNAcb1-3Galb1-4GlcNAcb1-3Galb1-4GlcNAcb1-6(Galb1-4GlcNAcb1-3Galb1-4GlcNAcb1-3Galb1-4GlcNAb1-2)Mana1-6(Galb1-4GlcNAcb1-3Galb1-4GlcNAcb1-3Galb1-4GlcNAcb1-2Mana1-3)Manb1-4GlcNAcb1-4(Fuca1-6)GlcNAcb-Sp24
564	Galb1-4GlcNAcb1-3Galb1-4GlcNAcb1-3GalNAca-Sp14
565	Galb1-4GlcNAcb1-3Galb1-4GlcNAcb1-6(Galb1-3)GalNAca-Sp14
566	Galb1-4GlcNAcb1-3Galb1-4GlcNAcb1-6(Galb1-4GlcNAcb1-3Galb1-4GlcNAcb1-3)GalNAca-Sp14
567	Neu5Aca2-3Galb1-4GlcNAcb1-3Galb1-4GlcNAcb1-3GalNAca-Sp14
568	GlcNAcb1-3Galb1-4GlcNAcb1-3GalNAca-Sp14
569	GlcNAcb1-3Galb1-4GlcNAcb1-6(Galb1-3)GalNAca-Sp14
570	GlcNAcb1-3Galb1-4GlcNAcb1-6(GlcNAcb1-3Galb1-4GlcNAcb1-3)GalNAca-Sp14
571	Neu5Aca2-3Galb1-4GlcNAcb1-3Galb1-4GlcNAcb1-6(Neu5Aca2-3Galb1-4GlcNAcb1-3Galb1-4GlcNAcb1-3)GalNAca-Sp14
572	Neu5Aca2-6Galb1-4GlcNAcb1-3Galb1-4GlcNAcb1-3GalNAca-Sp14
573	GlcNAcb1-3Galb1-4GlcNAcb1-3Galb1-4GlcNAcb1-3GalNAca-Sp14
574	Galb1-4GlcNAcb1-3Galb1-3GalNAca-Sp14
575	Neu5Aca2-3Galb1-4GlcNAcb1-3Galb1-4GlcNAcb1-6(Galb1-3)GalNAca-Sp14
576	Neu5Aca2-6Galb1-4GlcNAcb1-3Galb1-4GlcNAcb1-6(Galb1-3)GalNAca-Sp14
577	Neu5Aca2-6Galb1-4GlcNAcb1-6(Galb1-3)GalNAca-Sp14
578	Neu5Aca2-3Galb1-4GlcNAcb1-3Galb1-4GlcNAcb1-2Mana1-6(Neu5Aca2-3Galb1-4GlcNAcb1-3Galb1-4GlcNAcb1-2Mana1-3)Manb1-4GlcNAcb1-4GlcNAcb-Sp12
579	GlcNAcb1-6(Neu5Aca2-3Galb1-3)GalNAca-Sp14
580	Neu5Aca2-6Galb1-4GlcNAcb1-3Galb1-4GlcNAcb1-6(Neu5Aca2-6Galb1-4GlcNAcb1-3Galb1-4GlcNAcb1-3)GalNAca-Sp14
581	Neu5Aca2-6Galb1-4GlcNAcb1-3Galb1-4GlcNAcb1-3Galb1-4GlcNAcb1-2Mana1-6(Neu5Aca2-6Galb1-4GlcNAcb1-3Galb1-4GlcNAcb1-3Galb1-4GlcNAcb1-2Mana1-3)Manb1-4GlcNAcb1-4GlcNAcb-Sp12
582	Neu5Aca2-3Galb1-4GlcNAcb1-3Galb1-4GlcNAcb1-3Galb1-4GlcNAcb1-2Mana1-6(Neu5Aca2-3Galb1-4GlcNAcb1-3Galb1-4GlcNAcb1-3Galb1-4GlcNAcb1-2Mana1-3)Manb1-4GlcNAcb1-4GlcNAcb-Sp12
583	Neu5Aca2-6Galb1-4GlcNAcb1-3Galb1-4GlcNAcb1-2Mana1-6(Neu5Aca2-6Galb1-4GlcNAcb1-3Galb1-4GlcNAcb1-2Mana1-3)Manb1-4GlcNAcb1-4GlcNAcb-Sp12
584	GlcNAcb1-3Fuca-Sp21
585	Galb1-3GalNAcb1-4(Neu5Aca2-8Neu5Aca2-8Neu5Aca2-3)Galb1-4Glcb-Sp21

Supplementary Table 2. Structure of the glycans present on CFG's printed array version 5.4

Primer name	Sequence 5’->3’	Use
EnvSia156-6His HiFi forward primer	TAA GCT TAG GAG GTT AAC ATA TGA GGC CGG AGA CAA TAC C	Primers for cloning of histidine tagged EnvSia156 into the expression vector pJS119K.
EnvSia156-6His HiFi reverse primer	TCA GTG ATG GTG ATG GTG ATG GGA GTG CCA GGG GCG TAT	
pJS119K forward primer	CAT CAC CAT CAC CAT CAC TGA GAA TTC AGC TTG GCT GTT TTG	
pJS119K reverse primer	ATG TTA ACC TCC TAA GCT TAA TTC	
EnvSia156 D14A forward primer-set 1	C TTG AAC GAG **<u>GCT</u>** AAC AGC CAT TAC	Primers for site directed mutagenesis of EnvSia156-D14A – set 1
EnvSia156 D14A reverse primer – set 1	GAG ATC CCC GGT ATT GTC	
EnvSia156 H134A forward primer – set 1	G AAC GAC GTG **<u>GCC</u>** TAT GTA AAT GAC G	Primers for site directed mutagenesis of EnvSia156-H134A – set 1
EnvSia156 H134A reverse primer – set 1	ATG CGC ATA GAG ATC CAG	
EnvSia156 D14A forward primer – set 2	G AAC GAA **<u>GCG</u>** AAC AGC CAC TAC TTC TAT ACC	Primers for site directed mutagenesis of EnvSia156-D14A – set 2 (synthesized)
EnvSia156 D14A reverse primer – set 2	CCG GGT ATT AGC CTG AAC GAA **<u>GCG</u>** AAC AGC	
EnvSia156 H134A forward primer – set 2	C GAC GTG **<u>GCG</u>** TAT GTT AAC GAT GAG CGT TG	Primers for site directed mutagenesis of EnvSia156-D14A – set 2 (synthesized)
EnvSia156 H134A reverse primer – set 2	GC ATG CGT ATG AAC GAC GTG **<u>GCG</u>** TAT GTT AAC	

Supplementary Table 3. Set of primers used to amplify EnvSia156 and generate its mutant derivatives. Mutated codons are shown bold and underlined.

Protein	Amino acid sequence	Nucleotide sequence
**EnvSia156 WT**	MRPETIPGISLNEDNSHYFYTRAGRRLS<br>AEEVDSWVDQYAGTQVKELMLCPNC<br>MRTSYASQVWDPIWRGYDPAGPDDQP<br>LLASLPPEERVAARGWIHTAWQLHQD<br>GIDIYARWIRRCRQRGISPWISMRMND<br>VHYVNDERCFLHSEFWRENPQLRRVPY<br>RFAEWTDRAFDYGRAEVREHHLKLIRE<br>LAARYDFDGLELDWMRFGFHFRPGYE<br>AEGAEILTAFTAEVRRLLDDWEKRRGH<br>KIHLGARIPSRPATALGLGMDAVTWAR<br>RGLVDMLVITPFWASAETDMPVEIWRQ<br>LLEGTGVTLAAGLEVLLRPYPDSPLFQT<br>NSLETVRGAAASLLDRGAQRIYLFNYM<br>DSQTAMEDLENYPTLLREIGSLETLAGK<br>PRRHVLTFADTWAPGEPRAIPLPATCRP<br>GEWRAFRLHTGPKPEPGEVIAALGIEGG<br>VAIGPETLEVRVNGELCAFLGLVDLSKP<br>RPDFPVYGFSVPLAAMRRGYNLIEVTA<br>RQELRFGWAEFLIRPWHSHHHHHH	ATGCGTCCGGAGACCATCCCGGGTATTAGCCTGAACGA<br>AGACAACAGCCACTACTTCTATACCCGTGCGGGTCGTCG<br>TCTGAGCGCGGAGGAAGTGGACAGCTGGGTTGATCAGT<br>ACGCGGGTACCCAAGTGAAGGAGCTGATGCTGTGCCCG<br>AACTGCATGCGTACCAGCTACGCGAGCCAGGTTTGGGA<br>CCCGATCTGGCGTGGTTATGATCCGGCGGGTCCGGATGA<br>TCAACCGCTGCTGGCGAGCCTGCCGCCGGAGGAACGTG<br>TTGCGGCGCGTGGTTGGATCCACACCGCGTGGCAGCTGC<br>ACCAAGACGGCATCGATATTTACGCGCGTTGGATTCGTC<br>GTTGCCGTCAGCGTGGTATCAGCCCGTGGATTAGCATGC<br>GTATGAACGACGTGCACTATGTTAACGATGAGCGTTGCT<br>TTCTGCACAGCGAGTTCTGGCGTGAAAACCCGCAACTGC<br>GTCGTGTGCCGTACCGTTTTGCGGAATGGACCGACCGTG<br>CGTTCGATTATGGCCGTGCGGAAGTGCGTGAACACCAC<br>CTGAAACTGATCCGTGAGCTGGCGGCGCGTTACGACTTT<br>GATGGTCTGGAACTGGACTGGATGCGTTTCGGCTTTCAC<br>TTCCGTCCGGGTTATGAGGCGGAAGGCGCGGAGATTCT<br>GACCGCGTTCACCGCGGAAGTTCGTCGTCTGCTGGACGA<br>TTGGGAGAAGCGTCGTGGTCACAAAATCCACCTGGGCG<br>CGCGTATTCCGAGCCGTCCGGCGACCGCGCTGGGTCTGG<br>GTATGGATGCGGTGACCTGGGCGCGTCGTGGTCTGGTG<br>GATATGCTGGTTATCACCCCGTTTTGGGCGAGCGCGGAG<br>ACCGATATGCCGGTTGAAATTTGGCGTCAGCTGCTGGAG<br>GGTACCGGCGTGACCCTGGCGGCGGGTCTGGAAGTTCT<br>GCTGCGTCCGTACCCGGACAGCCCGCTGTTTCAAACCAA<br>CAGCCTGGAAACCGTGCGTGGTGCGGCGGCGAGCCTGC<br>TGGATCGTGGCGCGCAGCGTATCTACCTGTTCAACTATA<br>TGGACAGCCAAACCGCGATGGAGGATCTGGAAAACTAT<br>CCGACCCTGCTGCGTGAGATTGGTAGCCTGGAAACCCTG<br>GCGGGCAAGCCGCGTCGTCATGTTCTGACCTTTGCGGAC<br>ACCTGGGCGCCGGGCGAGCCGCGTGCGATCCCGCTGCC<br>GGCGACCTGCCGTCCGGGTGAATGGCGTGCGTTCCGTCT<br>GCATACCGGTCCGAAACCGGAGCCGGGCGAAGTGATTG<br>CGGCGCTGGGTATTGAGGGTGGCGTTGCGATTGGCCCG<br>GAGACCCTGGAAGTGCGTGTTAACGGTGAACTGTGCGC<br>GTTCCTGGGCCTGGTGGACCTGAGCAAGCCGCGTCCGG<br>ATTTTCCGGTGTACGGTTTTAGCGTTCCGCTGGCGGCGA<br>TGCGTCGTGGCTATAACCTGATCGAGGTTACCGCGCGTC<br>AGGAACTGCGTTTTGGTTGGGCGGAATTCCTGATTCGTC<br>CGTGGCACAGCCACCACCACCACCACCACTGA
**EnvSia156 D14A**	MRPETIPGISLNE**A**NSHYFYTRAGRRL<br>…	…CATCCCGGGTATTAGCCTGAACGAA**GCG**ACAGCCAC<br>TACTTCTATACCCGTGCGGGTCGTCGTCT…
**EnvSia156 H134A**	…GISPWISMRMNDV**A**YVNDERCFLHS<br>EF…	…TTAGCATGCGTATGAACGACGTG**GCG**TATGTTAACG<br>ATGAGCGTTGCT…

Supplementary Table 4. Amino acid and nucleotide sequences of the synthesized EnvSia156 wild type and mutant derivatives. For both mutants, only the sequences immediately upstream and downstream of the mutated residues /codons (in bold) are shown.
