# Supplementary material for: Inverting family GH156 sialidases define an unusual catalytic motif for glycosidase action
Source: Nat Commun. 2019 Oct 23;10:4816. doi: 10.1038/s41467-019-12684-7 (PMC6811678; doi:10.1038/s41467-019-12684-7)
Supplement: Supplementary file 2 — Reporting Summary [file 41467_2019_12684_MOESM2_ESM.pdf]

nature research

Corresponding author(s): Gideon Davies and Chris Taron

Last updated by author(s): Sep 19, 2019

Reporting Summary

Nature Research wishes to improve the reproducibility of the work that we publish. This form provides structure for consistency and transparency in reporting. For further information on Nature Research policies, see Authors & Referees and the Editorial Policy Checklist .

Statistics

For all statistical analyses, confirm that the following items are present in the figure legend, table legend, main text, or Methods section.

n/a	Confirmed	
☐	☒	The exact sample size (*n*) for each experimental group/condition, given as a discrete number and unit of measurement
☐	☒	A statement on whether measurements were taken from distinct samples or whether the same sample was measured repeatedly
☐	☒	The statistical test(s) used AND whether they are one- or two-sided<br>*Only common tests should be described solely by name; describe more complex techniques in the Methods section.*
☒	☐	A description of all covariates tested
☒	☐	A description of any assumptions or corrections, such as tests of normality and adjustment for multiple comparisons
☒	☐	A full description of the statistical parameters including central tendency (e.g. means) or other basic estimates (e.g. regression coefficient) AND variation (e.g. standard deviation) or associated estimates of uncertainty (e.g. confidence intervals)
☐	☒	For null hypothesis testing, the test statistic (e.g. *F*, *t*, *r*) with confidence intervals, effect sizes, degrees of freedom and *P* value noted<br>*Give P values as exact values whenever suitable.*
☒	☐	For Bayesian analysis, information on the choice of priors and Markov chain Monte Carlo settings
☒	☐	For hierarchical and complex designs, identification of the appropriate level for tests and full reporting of outcomes
☒	☐	Estimates of effect sizes (e.g. Cohen's *d*, Pearson's *r*), indicating how they were calculated

Our web collection on statistics for biologists contains articles on many of the points above.

Software and code

Policy information about availability of computer code

Data collection	LSR II flow cytometer (BD Biosciences) was used for flow cytometry data acquisition.
Data analysis	Microsoft Excel (v14.5.1), GraphPad Prism (v6) and Origin (2018b) were used for data analysis and representation. FlowJo (v10.0) was used for flow cytometry data analysis.

For manuscripts utilizing custom algorithms or software that are central to the research but not yet described in published literature, software must be made available to editors/reviewers. We strongly encourage code deposition in a community repository (e.g. GitHub). See the Nature Research guidelines for submitting code & software for further information.

Data

Policy information about availability of data

All manuscripts must include a data availability statement. This statement should provide the following information, where applicable:

- Accession codes, unique identifiers, or web links for publicly available datasets
- A list of figures that have associated raw data
- A description of any restrictions on data availability

The authors declare that the data supporting the findings of this study are available within the paper and its supplementary information file. Crystallographic coordinates and structure factors have been deposited in the Protein Data Bank under accession codes 6RZD (apo), 6S00 (Neu5Ac complex), 6S04 (Neu5Gc complex), 6S0E (DANA complex) and 6S0F (KDN complex). Further details and information are available from the corresponding author upon request.

Field-specific reporting

Please select the one below that is the best fit for your research. If you are not sure, read the appropriate sections before making your selection.

Life sciences study design

All studies must disclose on these points even when the disclosure is negative.

Sample size	No sample size calculation was performed. Three experimental replicates were performed in order to show standard deviations for flow cytometry experiments.
Data exclusions	No data were excluded.
Replication	Flow cytometry data were confirmed with three experimental replications performed on different dates.
Randomization	No randomization.
Blinding	No blinding was performed.

Reporting for specific materials, systems and methods

We require information from authors about some types of materials, experimental systems and methods used in many studies. Here, indicate whether each material, system or method listed is relevant to your study. If you are not sure if a list item applies to your research, read the appropriate section before selecting a response.

Materials & experimental systems

n/a	Involved in the study	
☐	☒	Antibodies
☐	☒	Eukaryotic cell lines
☒	☐	Palaeontology
☒	☐	Animals and other organisms
☒	☐	Human research participants
☒	☐	Clinical data

Methods

n/a	Involved in the study	
☒	☐	ChIP-seq
☐	☒	Flow cytometry
☒	☐	MRI-based neuroimaging

Antibodies

Antibodies used	Lectins:<br>Biotinylated Sambucus nigra lectin (SNA) (Vector Labs, B-1305)<br>Biotinylated Maackia amurensis lectin II (MAL II) (Vector Labs, B-1265)<br>Secondary streptavidin:<br>Streptavidin-Alexa Fluor 647 (Thermo Fisher Scientific, S21374)
Validation	All antibodies were used for applications validated by antibody suppliers per quality assurance provided by each supplier.

Eukaryotic cell lines

Policy information about cell lines

Cell line source(s)	K562 cells were obtained from ATCC
Authentication	No authentication was performed but ATCC cell lines are authenticated and cells were used at low passage number
Mycoplasma contamination	K562 cells tested negative for mycoplasma contamination by the MycoAlert PLUS mycoplasma test kit (Lonza, LT07-710)
Commonly misidentified lines (See ICLAC register)	No commonly misidentified cells were used in this study

Flow Cytometry

Plots

Confirm that:

- [x] The axis labels state the marker and fluorochrome used (e.g. CD4-FITC).
- [x] The axis scales are clearly visible. Include numbers along axes only for bottom left plot of group (a 'group' is an analysis of identical markers).
- [x] All plots are contour plots with outliers or pseudocolor plots.
- [x] A numerical value for number of cells or percentage (with statistics) is provided.

Methodology

Sample preparation	K562 cells (ATCC) were grown in RPMI 1640 medium (Corning, MT10040CV) + 10% heat-inactivated fetal bovine serum (heat inactivated) (Corning, 35016CV) + 1% penicillin/streptomycin (Fisher Scientific, sv30010). K562 cells were resuspended in normal growth media and cells were distributed 300,000 cells/well into a V-bottom 96 well plate (Fisher Scientific, 0720096). Sialidases or PBS were added to respective wells to final concentrations of 4.25 µM (EnvSia156), 0.2 µM (Vibrio cholerae nanH sialidase), or equivalent volume PBS as a control. Cells were incubated with sialidase for 2 h at 37 °C, 5% CO2. Cells were then pelleted by centrifugation at 500 x g for 5 min. Supernatant was removed and replaced with 200 µL of biotinylated lectins at 10 µg/mL in cold PBS + 0.5% BSA (Sigma A9647-100G), lectins: biotinylated Sambucus nigra lectin (SNA) (Vector Labs, B-1305), or biotinylated Maackia amurensis lectin II (MAL II) (Vector Labs, B-1265). Cells were incubated with lectins for 30 min at 4 °C, followed by three washes (centrifugation at 500 x g for 5 min, removal of supernatant, and resuspension in cold PBS + 0.5% BSA). After the final wash, cells were resuspended in 2.5 µg/mL streptavidin-Alexa Fluor 647 (Thermo Fisher Scientific, S21374) in cold PBS + 0.5% BSA and incubated at 4 °C for 15 minutes. Three more washes were performed as described previously, and the cells were resuspended in PBS + 0.5% BSA containing 100 nM Sytox Green (Thermo Fisher Scientific, S7020)
Instrument	LSR II flow cytometer (BD Biosciences)
Software	Data collection and gating was performed in FlowJo v10.0. Data was plotted with GraphPad Prism v6
Cell population abundance	Example percent abundances are shown in the supplementary gating figure. The majority of the count fell in the K562 population as gated by SSC-A/FSC-A, of these cells >90% were singlet as determined by the FSC-A/FSC-H gating. For unstained or secondary only stained cells, >95% of isolated single cells were live, for lectin stained cells 50-90% of singlet cells were live due to cytotoxic effects of lectin binding during staining.
Gating strategy	Gating was performed around the K562 population in the SSC-A/FSC-A channel, followed by gating on single cells (FSC-H/FSC-A). Live singlet K562 cells were then isolated by gating for Sytox green-negative cells in the FITC laser channel.

- [x] Tick this box to confirm that a figure exemplifying the gating strategy is provided in the Supplementary Information.
